# Communication about environmental health risks: A systematic review

**DOI:** 10.1186/1476-069X-9-67

**Published:** 2010-11-01

**Authors:** Donna Fitzpatrick-Lewis, Jennifer Yost, Donna Ciliska, Shari Krishnaratne

**Affiliations:** 1 Effective Public Health Practice Project, McMaster University 1685 Main Street West, Hamilton, Ontario Canada; 2 School of Nursing, McMaster University 1200 Main Street West, Hamilton, Ontario Canada

## Abstract

**Background:**

Using the most effective methods and techniques for communicating risk to the public is critical. Understanding the impact that different types of risk communication have played in real and perceived public health risks can provide information about how messages, policies and programs can and should be communicated in order to be most effective. The purpose of this systematic review is to identify the effectiveness of communication strategies and factors that impact communication uptake related to environmental health risks.

**Methods:**

A systematic review of English articles using multiple databases with appropriate search terms. Data sources also included grey literature. Key organization websites and key journals were hand searched for relevant articles. Consultation with experts took place to locate any additional references.

Articles had to meet relevance criteria for study design [randomized controlled trials, clinical controlled trials, cohort analytic, cohort, any pre-post, interrupted time series, mixed methods or any qualitative studies), participants (those in community-living, non-clinical populations), interventions (including, but not limited to, any community-based methods or tools such as Internet, telephone, media-based interventions or any combination thereof), and outcomes (reported measurable outcomes such as awareness, knowledge or attitudinal or behavioural change). Articles were assessed for quality and data was extracted using standardized tools by two independent reviewers. Articles were given an overall assessment of strong, moderate or weak quality.

**Results:**

There were no strong or moderate studies. Meta-analysis was not appropriate to the data. Data for 24 articles were analyzed and reported in a narrative format. The findings suggest that a multi-media approach is more effective than any single media approach. Similarly, printed material that offers a combination of information types (i.e., text and diagrams) is a more effective than just a single type, such as all text. Findings also suggest that factors influencing response to risk communications are impacted by personal risk perception, previous personal experience with risk, sources of information and trust in those sources.

**Conclusions:**

No single method of message delivery is best. Risk communication strategies that incorporate the needs of the target audience(s) with a multi-faceted delivery method are most effective at reaching the audience.

## Background

The Effective Public Health Practice Project (EPHPP) prepared this review for the National Collaborating Centre for Methods and Tools (NCCMT) as part of their joint "Small Drinking Water Project" of the National Collaborating Centres for Public Health. The topic for the review was informed by the National Collaborating Centre for Environmental Health's "Needs, Gaps, and Opportunities Assessment" report, which initially identified methods and techniques for communication regarding boil water advisories as a highly important topic for practitioners [1,2]. However, the preliminary search revealed very few studies involving boiled water advisories, so the search was broadened to include communication of environmental health risks.

Effective communication about risk with the public and the media has an essential role within the public health system. Numerous existing and emerging environmental health risks face the public on a daily basis. Over the past several years, global populations have endured many environmental health threats, ranging from natural disasters and bioterrorism to viral outbreaks; communication about these threats has played a vital role on affected populations. The availability or lack of information regarding Hurricane Katrina, for example, played a clear role in determining how people chose to react to the disaster [3]. Additionally, effective risk communication can impact the public's perception of and trust in public health authorities at the regional, national and international level [4].

Effective communication is part of the risk analysis process and is necessary for management of information and opinion related to real and perceived hazards [5]. It is essential to inform the public in ways that do not create undue apathy, complacency, or overconfidence while not creating undue stress or alarm [6]. Systematic information delivery to the public is important for ensuring clear communication, enhancing understanding of risk and increasing transparency of risk-analysis for decision making. Effective information dissemination approaches are also important for eliciting desired outcomes, whether increased awareness or attitudinal or behavioural change [7].

An underlying goal of risk communication is to provide useful, relevant and accurate information in an understandable language and format for a particular audience or risk group [1,2]. This information may include the nature of the risk and potential benefits, uncertainties, rationale for action and strategies for managing risk. Risk communication strategies may vary across emergency and non-emergency situations, and may require local, regional, national or international responses [2].

The objective of this systematic review is to identify the effectiveness of communication strategies for environmental health risk, and factors that impact communication uptake. An increased understanding of the effectiveness of risk communication strategies can provide useful information about how policies and programs can and should be implemented for effectiveness. It can also provide information to avoid pitfalls and miscommunication in the future.

## Methods

### Search Methods

The search for relevant articles was conducted on several databases, including: MEDLINE and Pre-MEDLINE, EMBASE, Cochrane Central Register of Controlled Trials, PsychINFO, Effective Public Health Practice Project Database, Sociological Abstracts, Applied Social Sciences Index, CSA Worldwide Political Science Abstracts, Web of Science and Science direct. Journals were searched from the date of their inception to November 30, 2009. There were no intentional restrictions on date and there were no restrictions on study design, however only references published in English were included. Search terms were adapted according to the requirements of individual databases for subject heading terminology and syntax. Expert input was used to narrow search terms and to elicit recommendations for article inclusion. Authors and the research librarian worked collaboratively to develop the list of search terms and refined search strategy. Search terms included: effective, evaluat*, evidence, impact, outcome*, best practice*, risk* and communication. For a complete list of search terms, see Additional file 1. The initial search yielded 14,155 potentially relevant articles. Grey literature was searched for and included if deemed appropriate by reviewers. Key organization websites and key journals were hand searched for relevant articles (see Additional file 2) for a list of hand-searched journals). Consultation with experts took place to locate any additional references.

### Data Collection and Analysis

#### Selection of Studies

Two independent reviewers initially screened articles for relevance. Of the 14,155 retrieved articles, 270 titles and abstracts were identified as potentially relevant. Articles that included disease transmission, chronic diseases, terrorism, substance use, crime, obesity, pharmacological, accidents, and disease related diagnostic risk communication were excluded. Any article included for relevance by either reviewer was considered potentially relevant and eligible for full text screening.

A total of 270 articles underwent full text screening. Two reviewers independently screened all of the articles for inclusion. Articles were included if they met the following criteria:

• primary study design: randomized controlled trials (RCTs), clinical controlled trials (CCTs), cohort analytic, cohort, any pre-post, interrupted time series, mixed methods, qualitative case studies or qualitative descriptive studies

• participants: the public (groups, communities and populations or individually delivered population studies, such as mail outs)

• interventions: included, but not limited to any community-based intervention (methods or tools), including but not restricted to Internet, telephone, media-based interventions or a combination thereof

• reported measurable outcomes: awareness, knowledge, attitude or behavioural change related to importance/impact of environmental health risks

**Figure 1:**
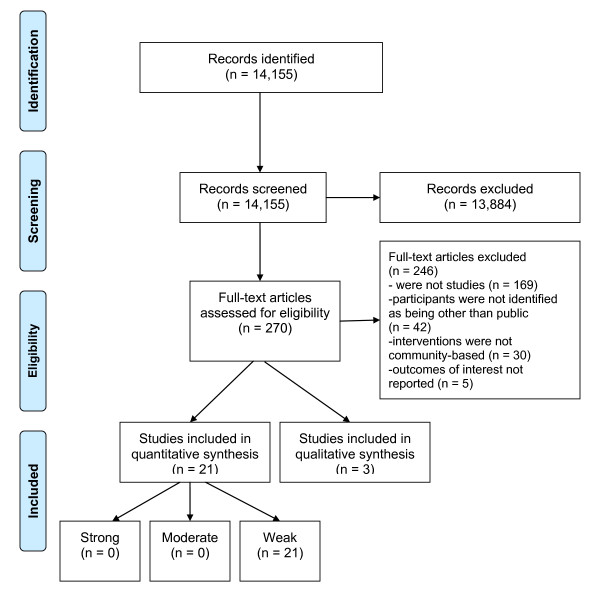
Flow Diagram

Conflicts were resolved through discussion. A total of 24 articles passed full text screening. A flow diagram (Figure 1) documents the selection process of articles included in the review. See Additional file 3 for a list of excluded studies.

#### Assessment of Risk of Bias in Included Studies

The 24 articles that passed full text screening were assessed for methodological quality by two independent reviewers. Quantitative and mixed-method studies were assessed using a tool that was developed and tested for primary studies in public health [8] (see Additional file 4). The tool is based on guidelines set out by Mulrow, Cook and Davidoff [9] and Jadad et al. [10] and includes six criteria (selection and allocation bias, blinding, confounders, data collection methods, and withdrawls and dropouts). The tool has been reviewed by experts in the field [11] and an accompanying dictionary is available on the EPHPP website: http://www.ephpp.ca.

Each of the 24 articles was rated on the six criteria as "strong," "moderate" or "weak," depending on characteristics reported in the study. Each study was given an overall assessment of strong, moderate or weak quality based on the total of each criterion rating. For a study to be rated as strong, none of the components could be rated as weak. A rating of moderate was achieved when only one component was rated weak. A rating of weak was given when two or more components were rated weak. The quality assessment results for relevant quantitative studies can be found in Table 1.

**Table 1:** Quality assessment results for relevant quantitative studies (n = 21)

Author/ Date	Selection Bias	Study Design	Confounders	Blinding	Data Collection Methods	Withdrawal/ Dropouts	GLOBAL RATING
Angulo et al. [21]	W	W	W	W	W	N/A	W

Atlas [26]	M	W	W	W	W	M	W

Blendon et al. [30]	W	W	W	W	W	N/A	W

Bord & O'Connor [22]	W	S	W	W	W	W	W

Burger et al. [23]	S	M	W	W	W	W	W

Burger & Waishwell [24]	S	W	W	W	W	N/A	W

Burnside et al. [31]	W	W	W	W	W	N/A	w

Connelly & Knuth [25]	W	W	W	W	W	N/A	W

Fox et al. [36]	M	W	W	W	W	N/A	W

Freimuth & Van Nevel (1993) [27]	M	M	W	W	W	W	W

Gutteling [13]	W	S	M	W	W	N/A	W

Johnson et al. [29]	M	S	W	W	W	W	W

Major [32]	M	M	W	W	W	N/A	W

Mileti & O'Brien [33]	W	W	M	W	W	W	W

Mulilis & Lippa [34]	W	S	W	W	W	W	W

Natter & Berry [18]	S	S	W	W	W	S	W

Predy et al. [17]	M	W	W	W	W	N/A	W

Rich & Conn [35]	W	S	M	W	W	W	W

Staats et al. [15]	W	M	W	W	S	M	W

Terpstra et al. [14]	W	M	W	W	W	W	W

Van Eijnd-hoven et al. [16]	M	W	W	W	W	W	W

KEY: W: Weak; M: Moderate; S: Strong; N/A: Not Applicable

Qualitative articles were reviewed according to criteria developed by Letts, Wilkins, Law, Stewart, Bosch, and Westoreland [12] (see Additional file 5). These criteria include, among other items, clarity of study purpose, auditability, credibility, transferability, dependability, and confirmability. The quality assessment results for relevant qualitative studies can be found in Table 2.

**Table 2:** Quality assessment results for qualitative relevant studies (n = 3)

STUDY	Eisenman **et al. **[3]	Blanchard **et al. **[19]	Perez-Lugo [20]
**STUDY PURPOSE**			

Was the purpose and/or research question stated clearly?	Yes	Yes	Yes

**LITERATURE**			

Was relevant background literature reviewed?	Yes	No	Yes

**STUDY DESIGN**			

What was the design?	Grounded theory	Qualitative description	Qualitative description

Was a theoretical perspective identified?	Yes	No	No

Method(s) used:	Interviews	Focus group	Interviews

**SAMPLING**			

Was the process of purposeful selection described?	Yes	No	Yes

Was sampling done until redundancy in data was reached?	Not addressed	Not addressed	Not addressed

Was informed consent obtained?	Yes	Not addressed	Not addressed

**DATA COLLECTION**			

**DESCRIPTIVE CLARITY: **Clear and complete description of site	No	No	No

Clear and complete description of participants	Yes	No	Yes

Role of researcher and relationship with participants	No	No	No

Identification of assumptions and biases of researcher	No	No	No

**PROCEDURE RIGOUR: **Was procedural rigour used in data collection strategies?	Not addressed	Yes	No

**DATA ANALYSIS**			

**ANALYTICAL RIGOUR: **Were data analyses inductive?	Yes	Yes	No

**ANALYTICAL RIGOUR: **Were findings consistent with and reflective of data?	Yes	Yes	Yes

**AUDITABILITY: **Was decision trial developed?	Yes	Not addressed	No

Was the process of analyzing the data described adequately?	Yes	Yes	No

**THEORETICAL CONNECTIONS: **Did a meaningful picture of the phenomenon under study emerge?	No	No	No

**OVERALL RIGOUR: **Was there evidence of the four components of trustworthiness?	Yes	Yes	Yes

Credibility	Yes	Yes	No

Transferability	Yes	Yes	No

Dependability	Yes	Yes	No

Confirmability	Yes	Yes	Yes

**CONCLUSIONS AND IMPLICATIONS: **Were the conclusions appropriate given the study findings?	Yes	Yes	Yes

DO The findings contribute to theory development and future practice/research?	Yes	Yes	Yes

Reviewers met to analyse their answers, discuss differences and reach a consensus for conflicting answers for all 24 articles. If a consensus over conflicts was not reached, they were forwarded to a third reviewer for resolution.

#### Data Extraction and Management

There were no strong or moderate studies; consequently all 24 weak articles were assessed for methodological quality and underwent data extraction using a standardized form. Data included authors, date of publication, objective, methods, participants, interventions, measurement instrument, and outcomes. See Additional file 6 (quantitative data extraction results) and Additional file 7 (qualitative data extraction results). During synthesis of the data, the use of theory in the included the 24 articles was further extracted.

### Data Synthesis

A meta-analysis was not appropriate with this group of studies due to heterogeneity in population, interventions and outcomes. All statistically significant and non-significant outcomes considered to be relevant to the review questions were reported. The results of the included studies are therefore presented in a narrative format to address the two research questions: 1) what is the effectiveness of risk communication strategies and 2) what factors impact risk communication uptake.

## Results

### Description of Studies

The 21 quantitative articles included in this review included randomized controlled trials, as well as cohort studies and interrupted time series, but the majority were one-time surveys or interviews. Samples representing U.S. adult men and women were most common; however four studies were conducted in the Netherlands [13-16] and one in both Canada [17] and the United Kingdom [18]. Total sample size ranged from 80 to 3,546. Each of the three qualitative articles used descriptive designs. Two [3,19] were conducted in the U.S., with one conducted in Puerto Rico [20].

The included studies were of various types of risks. A number of articles considered those associated with food consumption [21-25] such fish, salmonella, and food irradiation. Others included environmental risks (asbestos; toxins; radon gas; chemical spills; hazardous technology) [13,16,26-29], natural disasters (floods; hurricanes; earthquakes) [3,14,15,30-34], risks associated with bioterrorism and emergency preparedness [19,35], and those associated with viruses/disease (influenza; hantavirus pulmonary syndrome (HPS); West Nile) [17,18,36].

Theory was explicitly used in only three of the included studies. Protection Motivation Theory provided a conceptual framework in the study by Mulilis and Lippa [34], while Gutteling also used Protection Motivation Theory, in addition to the Theory of Planned Behaviour, and Social Cognitive Theory to guide selection of measurement tools [13]. The only study to actually test a theory (Social Cognitive Theory) was Major [32].

### Methods of Risk Communication

Print information [13,16,22-25,29] and media approaches [15,17,21,26-28,33,35] were the most frequently used method of risk communication. One study compared print information versus in-person/verbal communication [23] while another compared in-person/verbal versus no communication [14]. The outcome measured most often in the articles was change in knowledge [13-16,22,23,26,29,36]. Behaviour was also a commonly measured outcome with both hypothetical [24,30] and actual behaviour considered [15,21,33,34,36]. A limited number of articles measured additional outcomes such as judgement and acceptance of risk communication [22]. Lastly, articles also included data on subjects learning needs [24,30] and preferences for method of risk communication [13,18-20,25,31,32,36].

### Risk of Bias in Included Studies

All the quantitative articles achieved a rating of weak. For many of the articles, the rating reflected a lack of methodological information provided, as opposed to information that indicated the rating was weak. As previously stated, only a few articles with randomized controlled or clinical controlled designs were included. Most studies were one-time interviews and surveys which, in general, used questionnaires that had been developed specifically for that one study and had not been tested for reliability or validity. In only one study was a valid and reliable data collection tool used [15]. The data collection methods were often self-response (paper/pencil) and there was a strong possibility of recall bias. It was challenging to determine how representative the samples were of target population. Blinding of the data collector was frequently unreported. There were also high levels of dropout and withdrawals in studies with follow-up. Levels of statistical significance were also often not reported.

The three qualitative articles were assessed using the Letts et al. tool [12]. This tool does not provide an overall score for methodological quality; however, the component scores lead the reviewers to determine the three qualitative papers were methodologically weak. All of the studies provided a review of relevant background literature and a justification of need for the study. One of the studies used a grounded theory study design [3] and two studies used qualitative description [19,20]. However, even the study identified as a grounded theory did not give details of philosophical underpinnings or related data sampling and analysis strategies. All of the studies provided some information about the selection of participants, but there was limited discussion of how participants were approached by researchers. The interviews were semi-structured. Interview and focus group results were coded and grouped under themes and sub-themes to structure a narrative and discussion of findings. All of the studies provided a general discussion about the characteristics of participants. In terms of overall rigour, two articles [3,19] met the four components of trustworthiness (credibility, transferability, dependability and confirmability).

### Effectiveness of Risk Communication Strategies

The risk communication strategies evaluated in the included quantitative articles fall into three broad categories: print information [13,16,22-25,29]; media approaches [15,17,21,26-28,33,35]; and contact with experts [14]. None of the included qualitative articles measured the effectiveness of communication strategies.

### Print Information

#### Brochures

Bord and O'Connor, a U.S. controlled clinical trial, explored the use of different structured formats to communicate information about food irradiation [22]. Women (N = 195) were presented with one of eight documents that explained and diagrammed the food irradiation process. The sample was restricted to women as they were deemed to be the major purchaser of household food. Some brochures used technical language and others contained non-technical language. One half of the sample was also given a detailed verbal presentation of the major arguments for and against food irradiation, and the other half was given a short history of the use of irradiated food. The outcomes of this study indicate that the use of technical language, non-technical language or information about the pros/cons of food irradiation had little impact on the respondents' judgment. Acceptance increased when respondents knew the history of prestigious people (e.g., astronauts) who used the process and that a number of reputable federal and international agencies approved of irradiation. Overall, people who were well-informed about the topic had higher levels of acceptance. Those who scored high in knowledge of food irradiation also had higher levels of education (Pearson's r = .0.26), less distrust (r = -.28), lower alienation (r = -.20), anti-tech scores (r = -.23) and less fear of radiation (r = -.21). A key finding was that trust greatly impacted acceptability. Trust was related to industry in general, the food irradiation business specifically, government regulatory industries and the science that says food irradiation is safe.

Burger et al.'s controlled clinical trial examined the efficacy of two different formats for communicating the risks of eating contaminated fish: a brochure and a classroom presentation [23]. The sample included pregnant women and other women of childbearing age (N = 96) in the Newark Bay area of New Jersey. The information presented in both formats was the same, but the classroom lesson was longer and each point was presented in more detail than in the brochure. Both formats used detailed diagrams and each was available in English or Spanish. Ninety-six percent of the women who heard the presentation understood the information, compared with 72% of those reading the brochure. Those who heard the lesson provided the correct answers more often than did those who read the same information in the brochure for 18 of the 20 questions asked (p < .001).

A randomized 2 × 2 post-test design was used in the Netherlands by Gutteling to examine the effectiveness of brochures outlining the risks and benefits of a new hazardous technology [a plant for the oxygen-free burning of polyvinyl chloride (PVC)] [13]. Participants in the intervention group (N = 383) received one of two brochures, whereas the control group (N = 125) did not receive a brochure. The brochures were "sourced" from the government and a private company. Both brochures contained identical information with the exception of the conclusions. One half of the brochures contained explicit conclusions about the risks and benefits, and the other half did not have explicit conclusions. The brochure with the explicit conclusions also contained 12 evaluative remarks within the overall text that were not present in the other version of the brochure. The groups receiving the information, regardless of source, showed a statistically significant positive difference in knowledge of technology (p < .001); attitude about the technology (p < .001) and assessed the benefits of PVC as higher (p < .001) compared with the control group. There was no significant difference between the group that received the information and the control in assessment of risk of the technology, feelings of insecurity, attitude toward establishing a plant in the neighbourhood or intentions to seek additional information. Brochures from the private company aroused more fear than did the brochures from the government (p < .05). No significant differences were found in any of the measured variables when comparing those who received a brochure with or without explicit conclusions.

#### Fact sheets

A one-time post-test design explored four presentation formats for fact sheets about fish [25]. The purpose of the study by Connelly and Knuth was to better understand factors that can influence how people understand and respond to risk-related information [25]. The study was framed around the risk to human health from eating chemically contaminated non-commercial fish from the Great Lakes. Eight thousand questionnaires were mailed to a sample of licensed fishers in all the Great Lake states, with 3536 questionnaires completed. Detailed information about the sample was not provided, however the authors noted that the sample was 87% male and the majority had at least high school education. Four presentation formats were used: comparisons between grade 5 and grade 11 reading levels; diagram with descriptive text versus text only; a commanding, authoritative tone vs. a cajoling, more conversational tone; and qualitative vs. quantitative information on a comparative risk ladder. Respondents were asked to indicate which format (a) presented the information most clearly and understandably; (b) helped the reader best understand the health risks or other factors; (c) stimulated the reader's intention to engage in a particular behaviour [details not provided in the study]; and (d) provided the reader with the information needed to make his/her own decision about fish consumption. The authors identified households of concern as women of childbearing age and anglers living in households with children under the age of 15, however not all outcome measures were provided for this group. For instance, the preferred reading level for the households of concern was not reported. Those with less than a high school education were more likely to chose the fact sheets prepared at a grade 5 reading level (p < .01) than were those with at least a high school education. The households of concern were more likely to choose the text/diagram combination (p < .01). Households of concern were also more likely to choose the quantitative ladder than were other households (p < .05). Seventy-nine percent of all respondents indicated that the cajoling tone best suited their information needs compared with the commanding tone (level of statistical significance not reported).

Burger and Waishwell used a one-time survey in the U.S. to gain insight into whether fact sheets advising the risk of eating contaminated fish were read and the main messages understood [24]. Participants were given a fact sheet and interviewed to determine their knowledge, the major message, to whom the fact sheet should be distributed and suggestions for ways to deliver the message to fishers specifically, as well as to all others who might eat the fish. The sample (N = 92) was mostly male (88%), white (63%) with an age range of 23 to 77 years. There were no significant racial differences in the major messages received. Fifty-seven percent indicated that everyone should get the fact sheet and 37% indicated that it should go to those whom it concerned (fishers, purchasers, those living by the contaminated river). Most (86% of African Americans, 81% of Caucasians) indicated that fish consumption should be limited by some people. Seventy-one percent of respondents indicated they felt there were ways to reduce the risk associated with contaminated fish--primarily through the reduction of consumption of contaminated fish. Additional desirable information thought to be important included how to get additional copies of fact sheets, more information on the levels of contamination in fish and information on risk levels and ecological pathways.

A randomized controlled trial (social experiment) was conducted by Johnson, Fisher, Smith, and Desvouges to test the sensitivity of people's responses to alternative presentations of the same information on radon risk [29]. The intervention placed radon detection monitors in 2300 homes, shared readings from the monitors and provided printed materials that outlined the meaning of the reading and what should be done. Booklets were developed containing the same information about radon, but the information was presented in different formats: quantitative versus qualitative or commanding versus cajoling. The experiment was structured so that one half of those with a radon reading below 1 picocuries of radon per litre (pCi/1) [a low radon reading] received a fact sheet outlining radon. One of the five other booklets was randomly assigned to everyone else in the study. Those with a reading above 1 (pCi/1) also received the Environmental Protection Agency (EPA) publication, A Citizen's Guide to Radon [37]. The control group (N = 250) did not receive any readings or printed material. Both the experimental group and the control group received a quiz with multiple choice answers designed to measure respondents' knowledge about radon, how to measure it and how to mitigate it. Baseline measures were taken to determine the level of knowledge about radon, recall of previous risk information (there had been widespread media coverage) and perceived risk. Baseline knowledge was low, with fewer than half the respondents correctly answering the questions. Recall of previous risk messages was also low; only half of the experimental group and one quarter of the comparison group recalled hearing or reading any information about radon. Perceived risk was low with both groups. Homeowners who received a single-page fact sheet did not improve their scores (from baseline) on the risk questions in the follow-up survey. The control group that did not receive any information had improved scores in their knowledge about radon. The study authors suggested this might be a result of selection bias or the comparison group members paying more attention to media reports following their involvement with the baseline survey. The booklets worked better than the fact sheets, but no single format appeared to be best for all categories of test questions. The authors did not provide statistical data to support these claims.

#### Mailed information: Letter and card

The Seveso Directive is a broad European initiative that requires (among other things) the public who are most likely to be affected by an industrial accident be informed of their risks and the best way to act in the event of an accident. In response to the directive, a Dutch research group conducted a controlled clinical trial to help identify what information formats would be best for target populations [16]. The primary information was contained in a letter signed by the mayor and the director of the plant, as well as a card with instructions on the correct behaviour in case of an emergency. This mailing went out to all residents (number not reported) within a certain geographical region near the site of the controversial industries. The secondary information was provided only to those (number not reported) who replied to the letter and checked a request box indicating an interest in additional information that was on both the letter and the card. The secondary information was delivered at a public meeting (number not reported) in both communities where a representative from the company and the local municipality responded to questions or requests. Measurements were taken only on the effects of the total campaign. Quantitative measurements were not taken or reported in one community. In the second community, face-to-face interviews were conducted with male or female main inhabitants of selected addresses, with both pre-test (N *= *167) and post-test (N *= *159) interviews conducted. Pre-test measures indicated that residents had a good general level knowledge of the risks posed by the plants and the potential impact of a chemical spill. The campaign had only a slight positive effect on that knowledge. At pre-test, however, the populations had little information about what to expect in an emergency situation (only 17% knew the correct meaning of the siren signal). Following the campaign, 76% of the participants knew what the siren signal meant compared to 17% who knew at baseline. At the six-month follow-up (N *= *73), the campaign effect for that variable had decreased to 44% (level of statistical significance not reported).

### Media Approaches

#### Mass media

An interrupted time series evaluated a campaign to increase awareness of and information about the nature, extent and seriousness of asbestos exposure. The aim of the study conducted by Friemuth and Van Nevel was to deliver information to a target group of manual labourers over the age of 50 who could not be individually identified [27]. Public service announcements were created to be disseminated via media, including radio and television. The print media were supplied with a kit that contained a press release, several magazine and newspaper PSAs and pamphlets written for lay audiences and mailed to city editors. For each of the three waves of the probability survey, approximately ~1500 personal interviews were conducted. The percentage of people who believed that they had been exposed to asbestos increased from 26% to 33% between the pre- and post-campaign surveys. The post-campaign level of knowledge of asbestos-associated illness risks increased from 58% pre-campaign to 67% (level of statistical significance not reported).

Staats, Wit, and Midden used a pre/post-test design to evaluate the effect of a mass media campaign for communicating the risk of greenhouse gas [15]. The study was conducted in the Netherlands. A representative sample of the Dutch population with respect to age and sex (N = 965) were given pre-test questionnaires. Over a two-month period, an intense mass media campaign (national television, national newspapers, and billboards) was used to increase public awareness of the nature and cause of the greenhouse gas effect, its consequences and possible ways of dealing with this environmental problem. The campaign included 36 commercials for television, 14 advertisements for the newspapers and magazines and billboard and poster displays in high visibility public spaces. A follow-up questionnaire measured the impact of the campaign (n = 704) did not notice any of the campaign elements; 32% noticed TV-spots, posters and/or billboards; 8% read the advertisements. Increased knowledge of the greenhouse effect was greatest in the group that had seen the television commercials and billboards and had read the advertisements (p < .03). There was no campaign effect on emotional concern or on perceived seriousness of the problem. The campaign effect on voluntary behaviour change was evident only in terms of the separate disposal of small chemical waste (p <. 0001).

#### Educational program

A one-time survey studied the impact of an educational program delivered through printed new releases, newsletter articles, newspaper and radio PSA, video news release, prewritten letters to the editor and brochures, in English and Spanish, on increased awareness of West Nile Virus [36]. Fox, Averett, Hansen, and Neuberger, administered a survey to individuals in Kansas (N = 516) in urban and rural households with listed telephone numbers [36]. The State Department of Health and Environment (with a private marketing firm) produced and disseminated a multimedia campaign to increase awareness of and educate about West Nile Virus. The primary messages were: apply insect repellent containing DEET; wear long sleeves and pants during dawn and dusk; eliminate repositories of standing water; and check and repair window screens. Knowledge was widespread but preventative behaviours were not. Television (88%), newspapers (72%) and word-of-mouth (65%) were the most frequently cited sources of information. A small percentage of respondents cited health professionals (8%) as sources of information (level of statistical significances not reported).

#### Radio

A post-test survey explored the effectiveness of a boil water order that was delivered via radio to residents in a community with a Salmonella typhimurium outbreak [21]. Angulo et al. selected a random sample (N = 120 households with 329 members) from 548 households on the municipal tax roster [21]. Many residents (31%) drank unboiled water after being informed about the order. Twelve of 14 people who developed diarrhea after the issuance of the boil water order reported drinking unboiled water after being informed about the order. The most common reasons were forgetting (44%), not believing the initial notification (25%) and not understanding that ice should be made with boiled water (17%). Many of the people who reported that they heard the boil water order did not appreciate the severity of the situation; the initial boil water order did not give reasons for its issuance and did not mention associated illness.

#### Web-based

Atlas examined the effectiveness and use of the web-based U.S. Toxic Release Inventory (TRI), an internationally recognized environmental information program that provides industrial chemical release and transfer data [26]. The data was gathered in a three-phase survey design. Participants (N = 1292) were recruited from two counties. Telephone interviews were conducted with adults selected through random-digit dialling. Phase 1 of the survey measured baseline knowledge of TRI information; Phase 2 (N = 974) measured whether the publication of 1999 of TRI data affected respondents' knowledge of TRI; in Phase 3 (N = 847) participants were re-interviewed after being sent information about the 1999 data plus a website address where they could obtain additional TRI information. In all three phases there was low recall of the TRI data or facilities. For instance, at Phase 2 only 2% of the respondents recalled TRI without prompting when asked to name the government program that gathers and publicly reports information about industry's releases of chemicals into the environment (level of statistical significance not reported).

#### Automated phone message

A controlled clinical trial was used by Rich and Conn to explore the use of a computerized system to alert the public of hazards [35]. The automated dialling and message system conveyed pre-emergency information. The authors worked with local authorities to add a "sheltering in place" message to a routine test of a call down system. A call down system is a computerized calling system to alert residents that they might be affected by a chemical spill. The system is tested periodically by actually calling people's homes. For the purposes of this experiment, the test call for emergency preparedness was issued giving the listener the option of getting more information on sheltering in place. The intervention group (N = 209) was sent a pre-test questionnaire, received the test call and received a post-test questionnaire. Control group 1 (n = 74) did not receive the test call but received the pre and post questionnaires. Control group 2 (n = 69) did not receive the test call and only received the post-test questionnaire. The post-test response rates were as follows: 26% for the intervention group, 33% for control group 1 and 98% for control group 2. In the event of an emergency, 71% of those who received the call knew the right agency to call compared with 11% of those who did not receive the test call (level of statistical significance not reported). Prior to the test call, only 20% of the 55 respondents said they had seen or heard of the instructions on how to shelter-in-place. After the call, 64% said they had seen or heard such instruction, and 77% of those said they had received the instruction through the test call. For those who received the test call, there was an improvement in the percent of respondents who named each step in the effective sheltering (the study authors said this was statistically significant but did not provide the p values). Following the intervention, there was a reduction in percentage of the test group who indicated they did not know what to do to shelter (from 46% to 20%), but no statistically significant (p values not reported) change in the proportion of control group 1 who indicated they did not know what to do.

The second study, Predy, Carney, and Edwards used a one-time survey design to determine the effectiveness of a recorded information line in communicating health risk during the emergence of a new disease, hantavirus pulmonary syndrome (HPS), and to study the accuracy of recall of information about the virus among the general public [17]. The sample (N = 740) was randomly selected through telephone numbers in Edmonton, Canada. Two percent of the people received their information from the recorded line, and more people remembered receiving their information through the news media, particularly television (74%) and newspaper (57%). Many reported that the line was busy when they tried to call. Forty-four percent of respondents who recalled hearing the information about HPS took action to clean up mouse droppings or otherwise prevent them from coming in contact with mice or their droppings. The authors indicated that the media got much of their information from the information line, although there were no data to support this claim.

### Contact with Experts

Terpstra, Lindell, and Gutteling evaluated the effects of a flood risk communication program in the Netherlands [14]. The pre/post-test quasi-experimental study design measured the effect of direct personal experience and vicarious experience to risk communication on changes in individuals' beliefs and attitudes toward flood risk. The sample (N = 80) was drawn from a group of candidates who had run unsuccessfully in previous elections to be the general administrator of the local water board. Workshop participants (n = 24) had a multi-session experiential workshop with experts on flood risk; focus group participants (*n *= 16) spent time discussing flood risk but without experts; the control group (n = 40) received no information. There were no statistically significant differences among the three information conditions in the pre-test scores, suggesting the three groups were equivalent at baseline. Seven perceptions were measured, including: increasing risk, dread of risk, belief that they know the risk, known to science/experts, personal control, trust of authorities and public support for the risk-reducing measures. The workshop produced statistically significant changes from the pre-test to the post-test on two of seven dimensions: decrease in perceived societal support (p ≤ .05) and increase in perceptions of personal control (p ≤ .05). The workshop group showed stable change perceptions for three dimensions: increasing risk (p ≤ .01), dread risk (p ≤ .01) and trust (p ≤ .01). The focus group showed lower levels of attitude polarization than expected, and the control group showed no significant degree of attitude polarization.

### Factors that can impact communication uptake

Six quantitative and three qualitative articles included in this review examined factors that impact risk communication uptake. These can be grouped into the following categories of factors: personal risk perception [18,30,34]; previous experience with risk [33]; sources of information [9,31]; and trust in the source of information [3,19,20].

#### Personal Risk Perception

Blendon et al. used a one-time survey design to examine the future evacuation preparedness of people in areas heavily impacted by Hurricanes Katrina and Rita [30]. The sample was drawn from the counties of East Baton Rouge Parish (n = 500), Harris County Texas (n = 505), Mississippi and Alabama, adjacent to counties near the Gulf that had been declared disaster areas (n = 501) and Dallas County Texas (n = 500). Dallas was used as a comparison area because although it was not damaged by the hurricanes, experts had originally expected it to be hit by Hurricane Rita. The other three counties were chosen because they were heavily impacted by one or both of the hurricanes, but were outside the main area of heavy damage where telephone communication was not possible. In hypothetical questions about a future natural disaster such as a hurricane or flood, the researchers asked respondents if they would evacuate if told to do so by a government official and if not, why they would not leave. The survey found a large proportion (19-33%) of respondents would not evacuate. Houston respondents were statistically significantly more likely not to evacuate (p < .05) than were respondents in Baton Rouge or Mississippi/Alabama. Respondents who said they would not leave gave these explanations:

• thought they would be safe at home (73-79%)

• thought that the hurricane and its aftermath would not be too bad (42-51%)

• need to protect property (20-31%)

• not able to get gas (16-29%)

• did not know where to go to be safe (11-21%)

• could not afford to leave (8-23%)

• tried but unable to leave (6-21%)

• did not want to leave pets (10-22%)

• physically could not leave (5-11%)

• caring for someone who could not leave (8-16%)

A second article, Mulilis and Lippa examined if negative threat appeals caused behaviour change for a sample of California homeowners [34]. People who agreed to participate were randomly assigned to one of the 16 cells of a 2 × 2 × 2 × 2 factorial design or a control group. Baseline questionnaires were sent to 243 participants; 154 questionnaires were returned. Questionnaires consisted of the Mulilis-Lippa Earthquake Preparedness Scale (MLEPS), demographic and earthquake history information, an experimental manipulation essay and manipulation checks (used to measure the effects of the essay). The essay consisted of four paragraphs of information about earthquakes. The participants read the essay meant to manipulate their beliefs around four dimensions: probability of a large earthquake, expected severity of earthquake damage, perceived effectiveness of earthquake preparedness and perceived capability of preparedness. These four conditions represented the four factors in a 2 × 2 × 2 × 2 between-subject factorial design. The subjects read one of 16 different combinations of the same four paragraphs - each paragraph dealing with low or high conditions for each of the four dimensions. Approximately five weeks later, follow-up questionnaires were sent to 145 of the 154 participants (representing those who could be located at home). Seventy nine percent (experimental group n = 87, control group n = 27) of the follow-up surveys were returned. The four manipulated variables all strongly influenced subjects' beliefs at the time the essays were read (p < .05). There were no gender differences in findings. Participants subjected to the high effectiveness condition exhibited greater preparedness behaviour when they read the high probability essays than when they read the low probability essays (p = .04). Negative threat-inducing persuasive messages influenced preparedness over a five-week period after they were read. Further, this increase in preparedness did not appear to be the result of completing the earthquake preparedness scale. The increased preparedness appeared to result from the experimental manipulations. The direct effects of the negative threat appeals on respondents (which were significant at the time the communication was read) diminished in intensity when an increase in preparedness behaviour was measured.

Natter and Berry's randomized controlled trial compared the relative and absolute forms of presenting risk information about influenza and the need for vaccination [18]. They investigated whether differences in people's estimates and evaluation of risk information change when they are presented with baseline data. This trial involved a two-factor between-subject design (with/without baseline × relative/absolute risk reduction). Participants were randomly allotted to one of four groups (55 per group). Using a fictitious scenario, participants (N = 220) were told that a severe influenza epidemic was about to hit Britain. Half the participants in both risk reduction formats were informed about the baseline risk with the sentence: "It is predicted that 10% of the adult population (i.e., 10 out every 100 adults) will be affected by the flu." The scenario also informed participants that the public had been advised to get vaccinated. Absolute risk reduction was communicated as: "With vaccination, the risk of being affected by the flu is 5% lower." Relative risk reduction was communicated by: "With vaccination, the risk of being affected by the flu is reduced by 50%." If the baseline risk was not communicated, numerical estimates of the risk of flu were significantly higher in both groups (with and without vaccination; p < .001). Participants given the absolute information were more satisfied (p < .05) with the information than those in the relative condition, but only when informed about the baseline. Participants given information in a relative format were more likely (p < .01) to indicate they would get vaccinated, but only if they were not informed of the baseline information.

Finally, Situational Communication Theory explores the relationship between public opinion and communication within the context of small, active group of people (called "publics") that develop around a specific issue. Major tested Situation Communication Theory using an interrupted time series design in the context of disaster communication, specifically an earthquake prediction for the New Madrid fault region [32]. The sample (N = 998) was adults living in Missouri or Illinois. A series of interview questions were used to determine the level of involvement for the respondent in a disaster situation according to the four types of publics based on situational theory: problem recognition, constrained recognition, fatalistics or routines. The problem-facing public recognizes the problem and believes that something can be done about it. The constrained public recognizes the problem but thinks nothing can be done. The fatalistic public does not recognize the problem and has the perception that very little can be done to affect the situation. The routine public does not recognize the problem nor perceive constraints.

The hypothesis being tested was that information seeking and processing are variable and the level of involvement differentiates high-involvement, problem-facing publics from other publics. The second interview furthered that analysis. Problem recognition was measured by asking, "How often do you stop to think about a major earthquake hitting the area?" Possible answers were: very often, sometimes, not often, almost never. Constraint recognition was measured by asking, "If you personally tried to do something to help protect yourself or your family from a major earthquake, do you think your efforts would make a lot of difference, some difference, not much difference or no difference at all?" The "constrained" and "problem-facers", in contrast with the "fatalistics", reported that the earthquake problem was personally important and that they sought information and clarification about the earthquake prediction's meaning from other people and the media. "Problem-facers" and "constrained" were more likely to send for government booklets about earthquake safety than were the "fatalistics". "Problem-facers" found television and radio news reports helpful in preparing them for the earthquake. Those identified as "constrained" talked with family members and made preparations for an earthquake. Unlike the "problem-facers", the "constrained" sought ways to reduce their perceived constraints by making preparations. "Problem-facing" were more likely to have reported involvement in the earthquake issue, to have spoken with others about earthquakes and to have done something to prepare for an earthquake.

#### Previous Experience with Risk

Mileti and O'Brien used a one-time survey to explore public response to risk information that was issued in the context of an on-going emergency (i.e., earthquake aftershocks) [33]. Two populations were studied, San Francisco (N = 734), which had a high media exposure, and Santa Cruz County (N = 918), where there had been substantially greater damage but lower media coverage. Three-quarters of the respondents reported using the media for information more because of the earthquake. As many as 83.8% of respondents reported hearing the aftershock warnings, but few were able to recall what had been said about the risk. The data consistently revealed that response to a protective warning response action was more likely in Santa Cruz County respondents, who experienced more damage than the residents of San Francisco. Experiencing loss in a disaster may make subsequent warnings more salient, thereby enhancing the likelihood of engaging in protective actions in response to the warning. The lack of mainshock damage may have created a normalization bias for non-victims: if they experienced little personal damage during the first earthquake, they would be biased toward thinking that they would not be impacted by the aftershocks. Study findings also indicated that perceived risk had a direct and positive impact on response to warnings with protective actions; warning information quality and quantity or reinforcement had a direct positive effect on response; pre-event hazard salience enhanced warning response both directly and indirectly; and, selected demographics could negatively constrain both perception of risk and warning response (e.g., being male or a member of a non-white ethic group).

#### Sources of Information

Burnside et al. considered a hypothetical evacuation order to residents in Greater New Orleans (N = 1207) [31]. This study used a one-time survey design. Respondents were asked: if public officials recommended an evacuation because of the threat of a hurricane this year, what would you most likely do: definitely evacuate, probably evacuate, probably not evacuate or definitely not evacuate. These four categories were combined into two categories: people who would evacuate and people who would not evacuate. People who viewed public officials' advice as an important source of information were more likely to evacuate (p <. 01). People who had viewed more visual images of hurricane damage in the past were more likely to evacuate (p < .01).

Blanchard et al., in a qualitative study, determined that the main source for information about the anthrax threat was the general media such as television and newspapers, as well as communication from the U.S. Centers for Disease Control (CDC) [19]. Some postal workers said that information was communicated by the United States Postal Service management team. Senate workers highlighted internal communication mechanisms as their main source of information. The majority of both postal and senate workers said that information, predominantly from health authorities at the CDC, was disseminated in a confusing and untimely way.

#### Trust in Sources of Information

All three qualitative articles considered how trust in sources of information can affect risk communication. Eisenman et al. examined factors affecting evacuation decisions among people affected by Hurricane Katrina [3]. The study focused on evacuation orders and issues of trust related to these orders. Semi-structured interviews were conducted with participants living in areas affected by Hurricane Katrina who received evacuation orders related to the hurricane. Participants (N = 58) were selected from the three largest evacuation shelters in the city of Houston; 93% had not evacuated until after Katrina hit landfall. The participants were asked to describe: (1) sources and understanding of information in the time period before the hurricane; (2) knowledge, perceptions and resources that influenced their evacuation behaviour before the hurricane's landfall; and (3) reflections on factors that would have altered their behaviour. The study highlighted issues of physical capacity, cognitive awareness, perception of risk, socio-cultural factors as well as trust when determining the factors that influenced a person's decision to stay or evacuate. In terms of trust, some participants felt that evacuation orders and communication from health authorities on the risks of the hurricane were disseminated in a deliberate way to benefit people living in affluent neighbourhoods. Participants recalled past hurricanes and did not remember extensive damage and flooding occurring as a result of those hurricanes. Many believed that authorities had "blown" the levees that were protecting residential areas from the rising waters to save wealthy neighbourhoods at the expense of the poorer, more marginalized neighbourhoods. They believed that authorities did not have their best interests in mind. This belief affected residents' trust in, and therefore reaction to risk communication messages. There was no discussion of the factors that influenced the participants' decisions in terms of their timing when evacuating the area.

Perez-Lugo sought to determine the role of the media in a community's coping strategy in the face of natural disasters [20]. Participants (N = 37) were residents living in areas of Puerto Rico that were recently affected by Hurricane Georges. The study used semi-structured interviews to obtain information from participants, the majority of whom said that the media played a key role in their experience with the hurricane by providing them with the most up to date information. Participants stated that the Internet, television or radio were their preferred medium; newspapers were mentioned as a secondary source of information. Many said that the media was their main source of information about the disaster and that the media influenced their coping strategies in the face of the disaster. It was noted that many participants were more motivated to use the media for emotional support, companionship and community ties than for updates on the hurricane. The media also provided supports to people that were isolated from society for various reasons.

Blanchard et al. examined risk communication strategies related to bioterrorism in the case of the anthrax threat in Washington, DC among postal (N = 36) and senate workers (N = 7) [19]. Using focus groups, the study determined that many postal workers did not trust the higher authorities from which risk communication information was disseminated. Many believed that authorities were too slow to evacuate the post office and to initiate nasal swab testing among postal workers. Others said that they felt that information from the CDC and the U.S. Postal Service management team was not disseminated to them in a timely manner because of their social class. Senate workers did not think that information from the CDC was consistent, and they felt that public health representatives were poorly organized. This led to feelings of mistrust.

## Discussion

Overall, the articles included in this review lacked methodological quality. In addition, information necessary to judge the methodological quality of the articles was lacking in both quantitative and qualitative articles. Although quality ratings were not possible for qualitative articles, the identified limitations of the included articles identified meant that none of the quantitative articles were judged as methodologically moderate or strong. It is recommended that future studies should attempt to overcome the methodological issues of the primary articles identified in this review.

Effective risk communication is challenging; no single approach works for all populations or for all environmental risk situations. Given the powerful influence of mass media on risk perception [38], it is noteworthy that the quantitative articles suggest that a multi-media approach is more effective than any single media approach. For example, a public service advertisement campaign that includes ads on radio, television, Internet and print media is more effective than a public service announcement campaign that targets a single media source. Similarly, printed material that offers a combination of information types (i.e., text and diagrams) is a more effective communication tool than just a single type, such as all text. Providing risk information verbally in a presentation or classroom setting is more effective than simply providing written brochures or fact sheets. In-person presentations provide the receiver with an opportunity to seek clarification and ask questions that may increase their understanding of the information.

One-time communications may be effective at increasing risk knowledge. However, the impact of the intervention diminishes with the passing of time. This suggests that when a warning method is for seldom-occurring real life events (e.g., chemical spill sirens), the public should receive regular information about the meaning of the warning.

Factors influencing response to risk communications are impacted by personal risk perception, previous personal experience with risk, sources of information and trust in those sources, and preferences for information. People who believe that they are not at personal danger are less likely to evacuate in the case of a hurricane warning. People who can identify the problem and feel that they have the power to do something about it tend to be more receptive to risk communication information. This is especially true if they have past experience in an emergency or natural disaster where they were not directly impacted by the event.

In addition, people who have personally experienced the impact of a natural disaster are more receptive to risk advisories and evacuation orders. There is some evidence that people who have experience with natural disasters but have not experienced damage to their own property or injury continue to believe that they are also protected against possible danger from impending disasters.

The important role that trust plays in risk communication uptake identified within conceptual literature is also supported by the results of this review [38-40]. People pay more attention to information delivered by credible sources. As a trusted and important source of risk information, people often turn to the media ahead of other sources, including public officials. For example, when the public perceive that public officials or plant management have been slow to respond or are perceived to have withheld important information, trust is diminished. This can have a serious impact on the effectiveness of communications from those sources. In addition to being tailored to the specific needs of the targeted population, trust in communication can be enhanced when it is presented to all the affected people in a timely manner, consistent, easy to understand, and comes from a trusted source.

Recommendations for risk communication plans in public health have previously been published [40] and receive empirical support in the following summary of recommendations to maximize effectiveness of risk communications as a result of this review:

• Ensure communication comes from a trusted source.

• Tailor communication for the audience.

• Build the content of messages with the strongest scientific evidence available.

• Incorporate text with visuals (pictures, diagrams) with qualitative and quantitative data for print materials.

• Disseminate information in the media through multiple sources.

• Deliver warning system notices for rare events on a regular and on-going basis.

• Develop communication strategies with the awareness that people make choices based on personal past experience with disasters.

• Ensure communication strategies are multi-modal and incorporate an opportunity for the public to have their questions and concerns addressed.

• Do not use automated phone call-in systems as a proxy for human interaction, however if used, ensure they are easily accessible.

## Conclusions

This systematic review of the effectiveness of communication tools and methods related to environmental health risks shows that the primary studies to date are of poor methodological quality. However, using the best available evidence, important lessons can be taken from these studies and applied to future research and the practice of risk communication. The included studies highlight that the impact or effectiveness of risk communication strategies is affected by personal risk-perception, trust in the source of information and previous personal experiences with emergencies. As well, the methods of delivering the message are important. People integrate messages more effectively when the message delivery incorporates personal interaction. No single method of message delivery is best. Risk communication strategies that incorporate the needs of the target audience with a multi-facetted delivery method are effective at reaching the largest audience.

## Abbreviations

EPHPP: Effective Public Health Practice Project; NCCMT: National Collaborating Centre for Methods and Tools; RCT: randomized controlled trial; CCT: clinical controlled trials; U.S.: United States; HPS: hantavirus pulmonary syndrome; PVC: polyvinyl chloride; EPA: Environmental Protection Agency; PSA: public service announcement; DEET: N,N-Diethyl-meta-toluamide; TRI: toxic release inventory; MLEPS: Mulilis-Lippa Earthquake Preparedness Scale; CDC: Center for Disease Control.

## Competing interests

The authors declare that they have no competing interests.

## Authors' contributions

DFL was involved in the screening of the retrieved articles, data analysis and drafting the manuscript; JY was involved in revision and preparation of the manuscript for publication; DC was involved in the conceptualization of the review, the screening of retrieved articles, data analysis and drafting of the manuscript; SK screening of articles and drafting manuscript. All authors read and approved the final manuscript.

## Supplementary Material

Additional file 1 **Search Strategy**. This file provides the complete list of search terms used to search MEDLINE, EMBASE, PsychINFO, CINAHL, and other databases. Click here for file

Additional file 2 **Hand Searched Journals**. This file provides a list of journals were hand-searched from the date of their inception to November 30, 2009 to locate additional articles for inclusion in this review. Click here for file

Additional file 3 **List of Excluded Articles**. This file provides the search a list of articles (in APA 5^th ^edition format) excluded from the review for the following reasons: 1) due to study design, e.g. (e.g., editorials, process or event descriptions; 2) participants were identified as being other than the public (e.g., health care professionals; 3) intervention(s) were not community-based; 4) outcomes of interest were not reported. Click here for file

Additional file 4 **Quality Assessment Tool for Quantitative Studies**. This file is contains the Effective Public Health Practice Project's (EPHPP) tool used to assess the methodological quality of quantitative primary studies that passed initial relevance screening for inclusion in this review. Click here for file

Additional file 5 **Quality Assessment Tool for Qualitative Studies**. This file contains the tool used to assess the methodological quality of qualitative primary studies that passed initial relevance screening for inclusion in this review. Click here for file

Additional file 6 **Data Extraction Results for Included Quantitative Studies**. This file contains the data extraction results for all 24 quantitative primary articles included in this review that were assessed for methodological quality. Data included authors, date of publication, objective, methods, participants, interventions, measurement instrument, and outcomes. Click here for file

Additional file 7 **Data Extraction Results for Included Qualitative Studies**. This file contains the data extraction results for all 3 qualitative primary articles included in this review that were assessed for methodological quality. Data included authors, date of publication, objective, methods, participants, interventions, measurement instrument, and outcomes. Click here for file
