# Supplementary material for: Communication about environmental health risks: A systematic review
Source: Environ Health. 2010 Nov 1;9:67. doi: 10.1186/1476-069X-9-67 (PMC2988771; doi:10.1186/1476-069X-9-67)
Supplement: Additional file 1 — Search Strategy. This file provides the complete list of search terms used to search MEDLINE, EMBASE, PsychINFO, CINAHL, and other databases. [file 1476-069X-9-67-S1.DOC]

**Additional File 1. Search Strategy**

Effectiveness/Lessons learned of methods of risk communication to the public

**MEDLINE** search strategy

| **Effectiveness/ lessons learned** | **Methods** | **Risk** | **Communication** | **Public** |
| --- | --- | --- | --- | --- |
| effective*  evaluat*  evidence  impact  outcome* best practice*  lesson* | method*  concept*  strateg*  tool*  model*  guideline*  format*  message*  framework*  principle*  plan*  theor* | risk*  threat*  emergen*  hazard*  preparedness  disaster*  crisis  crises  terrorism  pandemic  outbreak*  epidemic* | communication/  mass media/  information dissemination/  communicat*.ti,ab | public  population*  communit*  consumer*  stakeholder*  societ*  NOT  patient*  chronic disease* |

Effectiveness/Lessons learned of methods of risk communication to the public

**EMBASE** Search strategy

| **Effectiveness/ lessons learned** | **Methods** | **Risk** | **Communication** | **Public** |
| --- | --- | --- | --- | --- |
| effective*  evaluat*  evidence  impact  outcome* best practice*  lesson* | method*  concept*  strateg*  tool*  model*  guideline*  format*  message*  framework*  principle*  plan*  theor* | risk*  threat*  emergen*  hazard*  preparedness  disaster*  crisis  crises  terrorism  pandemic  outbreak*  epidemic* | interpersonal communication/  mass medium/  mass communication/  information dissemination/  communicat*.ti,ab | public  population*  communit*  consumer*  stakeholder*  societ*  NOT  patient*  chronic disease* |

Effectiveness/Lessons learned of methods of risk communication to the public

**PsycINFO** Search strategy

| **Effectiveness/ lessons learned** | **Methods** | **Risk** | **Communication** | **Public** |
| --- | --- | --- | --- | --- |
| effective*  evaluat*  evidence  impact  outcome* best practice*  lesson* | method*  concept*  strateg*  tool*  model*  guideline*  format*  message*  framework*  principle*  plan*  theor* | risk*  threat*  emergen*  hazard*  preparedness  disaster*  crisis  crises  terrorism  pandemic  outbreak*  epidemic* | communication/  mass media/  persuasive communication/  information dissemination/  communicat*.ti,ab | public  population*  communit*  consumer*  stakeholder*  societ*  NOT  patient*  chronic disease* |

Effectiveness/Lessons learned of methods of risk communication to the public

**CINAHL** Search strategy

| **Effectiveness/ lessons learned** | **Methods** | **Risk** | **Communication** | **Public** |
| --- | --- | --- | --- | --- |
| effective*  evaluat*  evidence  impact  outcome* best practice*  lesson* | method*  concept*  strateg*  tool*  model*  guideline*  format*  message*  framework*  principle*  plan*  theor* | risk*  threat*  emergen*  hazard*  preparedness  disaster*  crisis  crises  terrorism  pandemic  outbreak*  epidemic* | communication/  communications media/  TI information dissemination  AB information dissemination  TI communicat*  AB communicat* | public  population*  communit*  consumer*  stakeholder*  societ*  NOT  patient*  chronic disease* |

Effectiveness/Lessons learned of methods of risk communication to the public

**Other databases**:

| **Effective** | **risk** | **communication** |
| --- | --- | --- |
| effective*  evaluat*  evidence  impact  outcome*  “best practice*”  lesson* | risk*  threat*  emergen* hazard*  preparedness  disaster*  crisis  crises  terrorism  pandemic  outbreak*  epidemic* | communicat*  disseminat*  mass media |
