# Supplementary material for: Communication about environmental health risks: A systematic review
Source: Environ Health. 2010 Nov 1;9:67. doi: 10.1186/1476-069X-9-67 (PMC2988771; doi:10.1186/1476-069X-9-67)
Supplement: Additional file 2 — Hand Searched Journals. This file provides a list of journals were hand-searched from the date of their inception to November 30, 2009 to locate additional articles for inclusion in this review. [file 1476-069X-9-67-S2.DOC]

**Additional File 2. Hand Searched Journals**

The following journals were searched for potential relevant studies:

- *Environmental Communication*
- *Stochastic Environmental Research and Risk Assessment*
- *Applied Environmental Education and Communication*
- *Environment International; Risk*
- *Risk Assessment; Health, Risk, and Society*
- *Risk, Issues in Health and Safety*
- *Risk Management and Healthcare Policy*
- *Journal of Applied Communication Research*
- *American Journal of Public Health*
- *American Journal of Health Promotion*
- *Canadian Journal of Public Health; Health Education and Behaviour*
- *Health Promotion International*
- Journal of Epidemiology and Community Health
