# Supplementary material for: Communication about environmental health risks: A systematic review
Source: Environ Health. 2010 Nov 1;9:67. doi: 10.1186/1476-069X-9-67 (PMC2988771; doi:10.1186/1476-069X-9-67)
Supplement: Additional file 3 — List of Excluded Articles. This file provides the search a list of articles (in APA 5th edition format) excluded from the review for the following reasons: 1) due to study design, e.g. (e.g., editorials, process or event descriptions; 2) participants were identified as being other than the public (e.g., health care professionals; 3) intervention(s) were not community-based; 4) outcomes of interest were not reported. [file 1476-069X-9-67-S3.DOC]

**Additional File 3. List of Excluded Articles**

**1) Articles Excluded: Due to Study Design, e.g. (e.g., editorials, process or event descriptions**

Abrams, D. B., Mills, S., & Bulger, D. (1999). Challenges and future directions for tailored communication research. *Annals of Behavioral Medicine*, *21*, 299-306.

Aedo, I., Diaz, P., Carroll, J. M., Convertino, G., & Rosson, M. B. (2009). End-user oriented strategies to facilitate multi-organizational adoption of emergency management information systems. *Information Processing & Management*, *46*, 11-21.

Albini, C. (2007). Toward "knowledge societies." Informing, communicating, and sharing. *Aggiornamenti Sociali*, *58*, 199-209.

Alder, G. S. (1997). Managing environmental uncertainty with legitimate authority: A comparative analysis of the Mann Gulch and Storm King Mountain Fires. *Journal of Applied Communication Research*, *25*, 98-114.

Alriksson, S. & Oberg, T. (2008). Conjoint analysis for environmental evaluation--a review of methods and applications. *Environmental Science & Pollution Research*, *15*, 244-257.

Anderson, A. (1991). Source strategies and the communication of environmental affairs. *Media, Culture & Society*, *13*, 459-476.

Anderson, C. (1998). Countdown to disaster. How to plan an effective disaster drill. *Emergency Medical Services*, *27*, 59-61.

Andrews, R. A., Austin, C., Brown, R., Chen, Y. Z., Engindeniz, Z., Girouard, R., et al. (2001). Sharing international experiences in disasters: Summary and action plan. *Prehospital & Disaster Medicine*, *16*, 42-45.

Andrulis, D. P., Siddiqui, N. J., & Gantner, J. L. (2007). Preparing racially and ethnically diverse communities for public health emergencies. *Health Affairs (Project Hope)*, *26*, 1269-1279.

Anonymous United States Congresses (2008). Strategies for risk communication. Evolution, evidence, experience. Proceedings of a symposium sponsored by the Society for Risk Analysis and the National Science Foundation. May 15-17, 2006. Montauk, New York, USA. *Annals of the New York Academy of Sciences*, *1128*, ix-xii.

Arias, E. H., Asai, Y., Chen, J. C., Cheng, H. K., Ishii, N., Kinugasa, T., et al. (2001). Sharing Pacific-Rim experiences in disasters: Summary and action plan. *Prehospital & Disaster Medicine*, *16*, 29-32.

Arya, N., Howard, J., Isaacs, S., McAllister, M. L., Murphy, S., Rapport, D., et al. (2009). Time for an ecosystem approach to public health? Lessons from two infectious disease outbreaks in Canada. *Global Public Health*, *4*, 31-49.

Attwater, R. & Derry, C. (2005). Engaging communities of practice for risk communication in the Hawkesbury Water Recycling Scheme. *Action Research*, *3*, 193-209.

Auld, M. E. (1990). Food risk communication: Lessons from the Alar controversy. *Health Education Research*, *5*, 535-543.

Avery, E. J. & Kim, S. (2009). Anticipating or precipitating crisis? Health agencies may not be heeding best practice advice in avian flu press releases. *Journal of Public Relations Research*, *21*, 187-197.

Baker, E. J. (1991). Hurricane Evacuation Behavior. *International Journal of Mass Emergencies and Disasters*, *9*, 287-310.

Baker, F. (1990). Risk Communication about environmental hazards. *Journal of Public Health Policy*, *11*, 341-359.

Baldwin, G. T., De Rosa, C., Howze, E. H., & Kreuter, M. W. (2004). Understanding wicked problems: A key to advancing environmental health promotion. *Health Education & Behavior*, *31*, 441-454.

Banerji, D. (2004). Reinventing mass communication: a World Health Organization tool for behavioral change to control disease. *International Journal of Health Services*, *34*, 15-24.

Beecher, N., Harrison, E., Goldstein, N., McDaniel, M., Field, P., & Susskind, L. (2005). Risk perception, risk communication, and stakeholder involvement for biosolids management and research. *Journal of Environmental Quality*, *34*, 122-128.

Benigeri, M. & Pluye, P. (2003). Shortcomings of health information on the Internet. *Health Promotion International*, *18*, 381-386.

Berry, T. R., Wharf-Higgins, J., & Naylor, P. J. (2007). SARS wars: An examination of the quantity and construction of health information in the news media. *Health Communication*, *21*, 35-44.

Bohm, G., Nerb, J., McDaniels, T., & Spada, H. (2001). Environmental risks – perception, evaluation and management: Introduction. *Research in Social Problems and Public Policy*, *9*, xi-xx.

Borda, J. L. & Mackey-Kallis, S. (2004). A model for crisis management. In D.P.Millar & R. L. Heath (Eds.), *Responding to crisis: A rhetorical approach to crisis communication* (pp. 117-137). Mahwah, NJ: Lawrence Erlbaum Associates Publishers.

Bostrom, A. (1994). Toward evaluation of effective risk communication. *Hazardous Substances & Public Health*, *3*, 7-8.

Bostrom, A. & Fischhoff, B. (2001). Communicating health risks of global climate change. *Research in Social Problems and Public Policy*, *9*, 31-56.

Bouye, K. E., Truman, B. I., Hutchins, S., Richard, R., Brown, C., Guillory, J. A., et al. (2009). Pandemic influenza preparedness and response among public-housing residents, single-parent families, and low-income populations. *American Journal of Public Health*, *99*, S287-S293.

Bradbury, J. A. (1994). Risk communication in environmental restoration programs. *Risk Analysis*, *14*, 357-363.

Bravata, D. M., McDonald, K. M., Szeto, H., Smith, W. M., Rydzak, C., & Owens, D. K. (2004). A conceptual framework for evaluating information technologies and decision support systems for bioterrorism preparedness and response. *Medical Decision Making*, *24*, 192-206.

Brenner, R. J. (2006). To err is human, to correct divine: The emergence of technology-based communication systems. *Journal of the American College of Radiology*, *3*, 340-345.

Briggs, D. J., Sabel, C. E., & Lee, K. (2009). Uncertainty in epidemiology and health risk and impact assessment. *Environmental Geochemistry and Health*, *31*, 189-203.

Browning, L. D. & Shetler, J. C. (1992). Communication in crisis, communication in recovery: A postmodern commentary on the Exxon Valdez disaster. *International Journal of Mass Emergencies and Disasters*, *10*, 477-498.

Budwig, N., Uzgiris, I. C., & Wertsch, J. V. (2000). Communication: An arena of development. In *Communication: An arena of development* (pp. xv, 242). Westport, CT: Ablex Publishing.

Burger, J. & Gochfeld, M. (2006). A framework and information needs for the management of the risks from consumption of self-caught fish. *Environmental Research*, *101*, 275-285.

Burger, J., Mayer, H. J., Greenberg, M., Powers, C. W., Volz, C. D., & Gochfeld, M. (2006). Conceptual site models as a tool in evaluating ecological health: The case of the Department of Energy's Amchitka Island nuclear test site. *Journal of Toxicology & Environmental Health Part A*, *69*, 1217-1238.

Cameron, L. D. & Chan, C. K. (2008). Designing health communications: Harnessing the power of affect, imagery, and self-regulation. *Social and Personality Psychology Compass*, *2*, 262-282.

Casman, E. A. & Fischhoff, B. (2008). Risk communication planning for the aftermath of a plague bioattack. *Risk Analysis*, *28*, 1327-1342.

Chapman, J. A. (2006). Review of managing communications in a crisis. *Leadership & Organization Development Journal*, *27*, 316-317.

Chess, C. (2001). Organizational theory and the stages of risk communication. *Risk Analysis*, *21*, 179-188.

Civan, A., Doctor, J. N., & Wolf, F. M. (2005). What makes a good format: Frameworks for evaluating the effect of graphic risk formats on consumers' risk-related behavior. *AMIA Annual Symposium Proceedings*, Annual Symposium Proceedings/AMIA Symposium, 927.

Clarke, L., Chess, C., Holmes, R., & O'Neill, K. M. (2006). Speaking with one voice: Risk communication lessons from the U.S. anthrax attacks. *Journal of Contingencies and Crisis Management*, *14*, 160-169.

Cohen, H. H., Cohen, J., Mendat, C. C., & Wogalter, M. S. (2006). Warning channel: Modality and media. In M.S.Wogalter (Ed.), *Handbook of warnings* (pp. 123-134). Mahwah, NJ: Lawrence Erlbaum Associates Publishers.

Comfort, L. K. & Haase, T. W. (2006). Communication, coherence, and collective action: The impact of Hurricane Katrina on communications infrastructure. *Public Works Management & Policy*, *10*, 328-343.

Coombs, W. T. (2006). Crisis management: A communicative approach. In C. H. Botan & V. Hazleton (Eds.), *Public Relations Theory II* (pp. 171-197). Mahwah, NJ: Lawrence Erlbaum Associates Publishers.

Cordova-Villalobos, J. A., Sarti, E., Arzoz-Padres, J., Manuell-Lee, G., Mendez, J. R., & Kuri-Morales, P. (2009). The influenza A(H1N1) epidemic in Mexico. Lessons learned. *Health Research Policy & Systems*, *7*, 21.

Cox, A., Guglielmetti, P., & Coulombier, D. (2009). Assessing the impact of the 2009 H1N1 influenza pandemic on reporting of other threats through the Early Warning and Response System. *Eurosurveillance,* *14,* 1-3.

Danczyk, P. A. (2007). *Intergovernmental interactions in threat preparedness and response: California's networked approach* (Doctoral dissertation). Retrieved from Proquest Dissertation Abstracts International (Publication No. AAT 3349170).

Day, T. G. (2003). The autumn 2001 anthrax attack on the United States Postal Service: The consequences and response. *Journal of Contingencies and Crisis Management*, *11*, 110-117.

De Marchi, B. (2003). Comments on Ortwin Renn's article 'Hormesis and risk communication': Considerations about uncertainity, ignorance and governance[comment]. *Human & Experimental Toxicology*, *22*, 25-29.

De Rosa, C. T., Pohl, H. R., Williams, M., Ademoyero, A. A., Chou, C. H., & Jones, D. E. (1998). Public health implications of environmental exposures. *Environmental Health Perspectives*, *106* Suppl 1, 369-378.

Driedger, S. M. & Eyles, J. (2003). Different frames, different fears: Communicating about chlorinated drinking water and cancer in the Canadian media. *Social Science & Medicine*, *56*, 1279-1293.

Duhe, S. (2008). Communicating Katrina: A resilient media. *International Journal of Mass Emergencies and Disasters*, *26*, 112-127.

Eysenbach, G. (2009). Infodemiology and infoveillance: Framework for an emerging set of public health informatics methods to analyze search, communication and publication behavior on the internet. *Journal of Medical Internet Research*, *11*, e11.

Ferreira, F. F. M., Guimaraes, F. R., Fumian, T. M., Victoria, M., Vieira, C. B., Luz, S., et al. (2009). Environmental dissemination of group A rotavirus: P-type, G-type and subgroup characterization. *Water Science and Technology*, *60*, 633-642.

Fisher, A. (1991). Risk communication challenges. *Risk Analysis*, *11*, 173-179.

Fitzpatrick, C. & Mileti, D. S. (1994). Public risk communication. In R.R.Davies & K. J. Tiemey (Eds.), *Disasters, collective behavior, and social organization* (pp.71-84) *.* Newark: University of Delaware Press.

Fox, C. R. & Irwin, J. R. (1998). The role of context in the communication of uncertain beliefs. *Basic and Applied Social Psychology*, *20*, 57-70.

Freimuth, V., Linnan, H. W., & Potter, P. (2000). Communicating the threat of emerging infections to the public. *Emerging Infectious Diseases*, *6*, 337-347.

Freimuth, V. S. (2006). Order out of chaos: The self-organization of communication following the anthrax attacks. *Health Communication*, *20*, 141-148.

Frewer, L. (2004). The public and effective risk communication. *Toxicology Letters*, *149*, 391-397.

Glik, D. C. (2007). Risk communication for public health emergencies. *Annual Review of Public Health*, *28*, 33-54.

Gong, G. & Dragga, S. (2008). "SARS" versus "atypical pneumonia": Inconsistencies in Hong Kong's public health warnings and disease-prevention campaign. In J. H. Powers & X. Xiao (Eds.), *The social construction of SARS: Studies of a health communication crisis* (pp. 53-68). Amsterdam, Netherlands: John Benjamins Publishing Company, Netherlands.

Granatt, M. (2004). On trust: Using public information and warning partnerships to support the community response to an emergency. *Journal of Communication Management*, *8*, 354-365.

Guiora, A. N. & Page, E. M. (2006). Comment: Lessons learned from "The Fifth Plague." *Case Western Reserve Journal of International Law*, *38*, 3-4.

Guo, Y. & Tang, K. (2004). From SARS outbreak to public following mind and media responsibility. *Zhuzhou Gong Xueyuan Xuebao/Journal of Zhuzhou Institute of Technology*, *18*, 106-107.

Hackett, A. J. (2008). Risk, its perception and the media: The MMR controversy. *Community Practitioner*, *81*, 22-25.

Halloran, R. (2007). Strategic communication. *Parameters*, *37*, 4-14.

Heath, R. L. (2004). Telling a story: A narrative approach to communication during crisis. In D. P. Millar & R. L. Heath (Eds.), *Responding to crisis: A rhetorical approach to crisis communication* (pp. 167-187). Mahwah, NJ: Lawrence Erlbaum Associates Publishers.

Heath, R. L. & Millar, D. P. (2004). A rhetorical approach to crisis communication: Management, communication processes, and strategic responses. In D. P. Millar & R. L. Heath (Eds.), *Responding to crisis: A rhetorical approach to crisis communication* (pp. 1-17). Mahwah, NJ: Lawrence Erlbaum Associates Publishers.

Heath, R. L. (2006). Best practices in crisis communication: Evolution of practice through research. *Journal of Applied Communication Research*, *34*, 245-248.

Izard, C. E. (2002). Translating emotion theory and research into preventive interventions. *Psychological Bulletin*, *128*, 796-824.

Johnson, B. L. (1987). Health risk communication at the agency for toxic substances and disease registry. *Risk Analysis*, *7*, 409-412.

Johnson, F. & Fisher, A. (1989). Conventional wisdom on risk communication and evidence from a field experiment. *Risk Analysis*, *9*, 209-213.

Johnson, H., Douglas, J., Bigby, C., & Iacono, T. (2009). "Maximising community inclusion through mainstream communication services for adults with severe disabilities": Corrigendum. *International Journal of Speech Language Pathology*, *11*, 339.

Johnson, H., Douglas, J., Bigby, C., & Iacono, T. (2009). Maximizing community inclusion through mainstream communication services for adults with severe disabilities. *International Journal of Speech Language Pathology*, *11*, 180-190.

Kalthoff, H. (2005). Practices of calculation: Economic representations and risk management. *Theory, Culture & Society*, *22*, 69-97.

Kapucu, N. (2006). Interagency communication networks during emergencies: Boundary spanners in multiagency coordination. *The American Review of Public Administration*, *36*, 207-225.

Kapucu, N., Augustin, M. E., & Garayev, V. (2009). Interstate partnerships in emergency management: Emergency management assistance compact in response to catastrophic disasters. *Public Administration Review*, *69*, 297-313.

Kasperson, R. E. (1986). Six propositions on public participation and their relevance for risk communication. *Risk Analysis*, *6*, 275-281.

Kasperson, R. E., Golding, D., & Tuler, S. (1992). Social distrust as a factor in siting hazardous facilities and communicating risks. *Journal of Social Issues*, *48*, 161-187.

Keeney, G. B. (2004). Disaster preparedness: What do we do now? *Journal of Midwifery and Women's Health*, *49*, 2-6.

Keune, H., Morrens, B., & Loots, I. (2008). Risk communication and human biomonitoring: Which practical lessons from the Belgian experience are of use for the EU perspective? *Environmental Health: A Global Access Science Source*, *7* (Suppl 1), S11.

Kim-Farley, R. J., Celentano, J. T., Gunter, C., Jones, J. W., Stone, R. A., Aller, R. D., et al. (2003). Standardized emergency management system and response to a smallpox emergency. *Prehospital & Disaster Medicine*, *18*, 313-320.

Kirn, A. (1999). Public communication in the case of risk. *Teorija in Praksa*, *36*, 944-956.

Kirschenbaum, A. (2005). Preparing for the inevitable: Environmental risk perceptions and disaster preparedness. *International Journal of Mass Emergencies and Disasters*, *23*, 97.

Kizer, K. W. (2000). Lessons learned in public health emergency management: Personal reflections. *Prehospital & Disaster Medicine*, *15*, 209-214.

Klauenberg, B. J. & Vermulen, E. K. (1994). Role for risk communication in closing military waste sites. *Risk Analysis*, *14*, 351-356.

Klinke, A. & Renn, O. (2002). A new approach to risk evaluation and management: Risk-based, precaution-based, and discourse-based strategies. *Risk Analysis*, *22*, 1071-1094.

Konheim, C. S. (1988). Risk communication in the real world. *Risk Analysis*, *8*, 367-373.

Kovats, R. S. & Ebi, K. L. (2006). Heatwaves and public health in Europe. *European Journal of Public Health*, *16*, 592-599.

Kovats, R. S. (2006). Heat waves and health protection. *British Medical Journal*, *333*, 314-315.

Krumkamp, R., Ahmad, A., Kassen, A., Hjarnoe, L., Syed, A. M., Aro, A. R., et al. (2009). Evaluation of national pandemic management policies — A hazard analysis of critical control points approach. *Health Policy*, *92*, 21-26.

Lee, T. R. (1986). Effective communication of information about chemical hazards. *Science of the Total Environment*, *51*, 149-183.

Leiss, W. (1995). "Down and Dirty": The use and abuse of public trust in risk communication. *Risk Analysis*, *15*, 685-692.

Leiss, W. (2004). Effective risk communication practice. *Toxicology Letters*, *149*, 399-404.

Lindhout, P. & Ale, B. J. M. (2009). Language issues, an underestimated danger in major hazard control? *Journal of Hazardous Materials*, *172*, 247-255.

Lipkus, I. M. (2007). Numeric, verbal, and visual formats of conveying health risks: Suggested best practices and future recommendations. *Medical Decision Making*, *27*, 696-713.

Liu, S., Huang, J. C., & Brown, G. L. (1998). Information and risk perception: A dynamic adjustment process. *Risk Analysis*, *18*, 689-699.

Lopez, A. T. & Vicente, M. (2008). *Some keys for improving the efficiency of environmental communication*. Barcelona, Spain: International Sociological Association.

Luber, G. & McGeehin, M. (2008). The health impacts of climate change: Climate change and extreme heat events. *American Journal of Preventive Medicine*, *35*, 429-435.

Lukens, J. G. (1978). Interethnic conflict and communicative distances. In H. Giles & R. Saint-Jacques (eds), *Language and Ethnic Relations* (pp. 147-158). Elmsford, NY: Pergamon Press.

Lule, J. (2006). Disasters and the news media. *International Journal of Media and Cultural Politics*, *2*, 348-353.

Lundqvist, L. J. (1982). Consultative mechanisms in Sweden's environment protection policy. *Environment International*, *7*, 379-387.

Marcus, A. C. (1999). New directions for risk communication research: A discussion with additional suggestions[comment]. *Journal of the National Cancer Institute*, *Monographs*, 35-42.

May, T. (2005). Public communication, risk perception, and the viability of preventive vaccination against communicable diseases. *Bioethics*, *19*, 407-421.

Mazis, M. B. & Morris, L. A. (1999). Channel. In M. S. Wogalter, D. M. DeJoy, & K. R. Laughery (Eds.), *Warnings and risk communication* (pp. 99-121). Philadelphia, PA: Taylor & Francis.

McCallum, D. B. (1995). Risk communication: A tool for behavior change. *NIDA Research Monograph*, *155*, 65-89.

Menon, K. & Goh, K. (2005). Transparency and trust: Risk communications and the Singapore experience in managing SARS. *Journal of Communication Management*, *9*, 375-383.

Mileti, D. S. & Peek, L. (2000). The social psychology of public response to warnings of a nuclear power plant accident. *Journal of Hazardous Materials*, *75*, 181-194.

Millar, D. P. & Heath, R. L. (Eds.) (2004). *Responding to crisis: A rhetorical approach to crisis communication*. Mahwah, NJ: Lawrence Erlbaum Associates Publishers.

Moghadas, S. M., Pizzi, N. J., Wu, J., & Yan, P. (2009). Managing public health crises: The role of models in pandemic preparedness. *Influenza and Other Respiratory Viruses*, *3*, 75-79.

Money, C. D., Jacobi, S., Penman, M. G., Rodriguez, C., De Rooij, C., & Veenstra, G. (2007). The ECETOC approach to targeted risk assessment; Lessons and experiences relevant to REACH. *Journal of Exposure Science and Environmental Epidemiology*, *17*, S67-S71.

Murray, V. & Goodfellow, F. (2002). Mass casualty chemical incidents – Towards guidance for public health management. *Public Health*, *116*, 2-14.

Needleman, C. (1987). Ritualism in communicating risk information. *Science, Technology, and Human Values*, *12*, 3-4.

Neuman, W. R. (1978). *The comparative analysis of national communications systems: Social control and social change*. Unpublished work.

Nicholson, P. J. (1999). Communicating health risk. *Occupational Medicine* (Oxford, England), *49*, 253-256.

Nicholson, P. J. (2000). Communicating occupational and environmental issues. *Occupational Medicine* (Oxford, England), *50*, 226-230.

Noel, F., Rondeau, E., & Sbeghen, J. (1998). Communication of risks: Organization of a methylmercury campaign in the Cree communities of James Bay, Northern Quebec, Canada. *International Journal of Circumpolar Health*, *57* (Suppl 1), 591-595.

Norris, F. H. & Stevens, S. P. (2007). Community resilience and the principles of mass trauma intervention. *Psychiatry*, *70*, 320-328.

Nsiah-Kumi, P. A. (2008). Communicating effectively with vulnerable populations during water contamination events. *Journal of Water and Health*, *6*, 63-75.

O'Malley, P., Rainford, J., & Thompson, A. (2009). Transparency during public health emergencies: From rhetoric to reality. *Bulletin of the World Health Organization*, *87*, 614-618.

Omenn, G. S. (2007). The risk assessment-risk management paradigm. In M. G. Robson & W. A. Toscano (Eds.), *Risk assessment for environmental health* (pp. 11-29). San Francisco, CA: Jossey-Bass.

Paton, D., Parkes, B., Daly, M., & Smith, L. (2008). Fighting the flu: Developing sustained community resilience and preparedness. *Health Promotion Practice*, *9,* 45S-53S.

Pearson, G. S. (2006). Public perception and risk communication in regard to bioterrorism against animals and plants. *Revue Scientifique et Technique*, *25*, 71-82.

Peters, E. (2008). Numeracy and the perception and communication of risk. *Annals of the New York Academy of Science, 1128,* 1-7.

Pidgeon, N. & Rogers-Hayden, T. (2007). Opening up nanotechnology dialogue with the publics: Risk communication or 'upstream engagement'? *Health, Risk & Society*, *9*, 191-210.

Pligt, J. & Midden, C. J. H. (1990). Chernobyl, four years later: Attitudes, risk management and communication. *Journal of Environmental Psychology*, *10*, 91-99.

Quarantelli, E. L. (1997). Ten criteria for evaluating the management of community disasters. *Disasters*, *21*, 39-56.

Quinn, S. C. (2008). Crisis and emergency risk communication in a pandemic: A model for building capacity and resilience of minority communities. *Health Promotion Practice*, *9,* 18-25.

Rasmussen, T. (2000). *Social theory and communication technology*. Aldershot, England: Ashgate.

Rother, H. A. (2006). Risk perception, risk communication, and the effectiveness of pesticide labels in communicating hazards to South African farm workers. *Dissertation Abstracts International, A: The Humanities and Social Sciences*, *66*, 3481-348A.

Saliou, P. (1994). Crisis communication in the event of a flu pandemic. *European Journal of Epidemiology*, *10*, 515-517.

Sandman, P. M. (2006). Crisis communication best practices: Some quibbles and additions. *Journal of Applied Communication Research*, *34*, 257-262.

Santos, S. L. (2007). Risk Communication. In M. G. Robson & W. A. Toscano (Eds.), *Risk assessment for environmental health* (pp. 463-486). San Francisco, CA: Jossey-Bass.

Sapriel, C. (2003). Effective crisis management: Tools and best practice for the new millennium. *Journal of Communication Management*, *7*, 348-355.

Savoia, E., Massin-Short, S. B., Rodday, A. M., Aaron, L. A., Higdon, M. A., & Stoto, M. A. (2009). Public health systems research in emergency preparedness: A review of the literature. *American Journal of Preventive Medicine*, *37*, 150-156.

Scanlon, J. & Frizzell, A. (1979). Old theories don't apply: Implications of communications in crises. *Disasters*, *3*, 315-319.

Seeger, M. W. (2006). Best practices in crisis communication: An expert panel process. *Journal of Applied Communication Research*, *34*, 232-244.

Sexton, K. (1992). The role of scientific research in risk assessment and risk management decisions. *Otolaryngology - Head and Neck Surgery*, *106*, 635-641.

Skanata, D. (1996). Risk presentation as basic element of risk communication. *Socijalna Ekologija*, *5*, 197-212.

Skertich, R. L. (2009). Action and information networks in disaster management. *Dissertation Abstracts International, A: The Humanities and Social Sciences*, *69*, 4498.

Slovic, P., Fischhoff, B., & Lichtenstein, S. (1982). Why study risk perception? *Risk Analysis*, *2*, 83-93.

Slovic, P. (1986). Informing and educating the public about risk. *Risk Analysis*, *6*, 403-415.

Slovic, P. (1987). Perception of risk. *Science*, *236*, 280-285.

Slovic, P., Peters, E., Finucane, M. L., & MacGregor, D. G. (2005). Affect, risk, and decision making. *Health Psychology*, *24*, S35-S40.

Spencer, R. C. & Lightfoot, N. F. (2001). Preparedness and response to bioterrorism. *Journal of Infection*, *43*, 104-110.

Stallings, R. A. (1995). *Promoting risk: Constructing the earthquake threat*. Hawthorne, NY: Aldine De Gruyter.

Stanciugelu, I. & Bratosin, S. (2009). Emergency communication and the challenge of messages. Theoretical insights. *Revista Romana de Comunicare si ^relatii publice*, *11*, 95-111.

Sterman, J. D. (2008). Risk communication on climate: Mental models and mass balance. *Science*, *322*, 532-533.

Strydom, P. (2008). Risk communication: World creation through collective learning under complex contingent conditions. *Journal of Risk Research*, *11*, 1-2.

Thomas, C. W., Vanderford, M. L., & Quinn, S. C. (2008). Evaluation and practice evaluating emergency risk communications: A dialogue with the experts. *Health Promotion Practice*, *9*, 5S-12S.

Thompson, K. M. (2002). Variability and uncertainty meet risk management and risk communication. *Risk Analysis*, *22*, 647-654.

Tinker, T. L., Zook, E., & Chapel, T. J. (2001). Key challenges and concepts in health risk communication: Perspectives of agency practitioners. *Journal of Public Health Management & Practice*, *7*, 67-75.

Toulmin, L. M. (1991). Emergency telecommunications: The national communications system in the United States. *Disasters*, *15*, 177-185.

Tuler, S., Webler, T., & Finson, R. (2005). Competing perspectives on public involvement: Planning for risk characterization and risk communication about radiological contamination from a national laboratory. *Health, Risk & Society*, *7*, 247-266.

van Baalen, P. J. & van Fenema, P. C. (2009). Instantiating global crisis networks: The case of SARS. *Decision Support Systems*, *47*, 277-286.

Vaughan, E. & Tinker, T. (2009). Effective health risk communication about pandemic influenza for vulnerable populations. *American Journal of Public Health*, *99*, S324-S332.

Veil, S. R., Littlefield, R. S., & Rowan, K. E. (2009). Dissemination as success: Local emergency management communication practices. *Public Relations Review*, *35*, 449-451.

Venette, S. (2009). Review of crisis communication and the public health. *Journal of Communication*, *59*, E22-E24.

Venette, S. J. (2006). Special section introduction: Best practices in risk and crisis communication. *Journal of Applied Communication Research*, *34*, 229-231.

Visschers, V. H. M., Meertens, R. M., Passchier, W. W. F., & De Vries, N. N. K. (2009). Probability information in risk communication: A review of the research literature. *Risk Analysis*, *29*, 267-287.

Vlek, C. A. (1995). Understanding, accepting and controlling risks: A multistage framework for risk communication. *European Review of Applied Psychology/Revue Europeenne de Psychologie Appliquee*, *45*, 49-56.

Wartella, E. & Middlestadt, S. (1991). The evolution of models of mass communication and persuasion. *Health Communication*, *3*, 205-215.

Wilde, G. J. S. (1993). Effects of mass media communications on health and safety habits: An overview of issues and evidence. *Addiction*, *88*, 983-996.

Williams, P. R. D. (2004). Health risk communication using comparative risk analyses. *Journal of Exposure Analysis and Environmental Epidemiology*, *14*, 498-515.

Wireman, J. R. & Long, G. C. (2001). Communicating risk in diverse communities. *Toxicology and Industrial Health*, *17*, 298-301.

Wogalter, M. S., DeJoy, D. M., & Laughery, K. R. (1999). Warnings and risk communication. In *Warnings and risk communication* (pp. xviii, 365). Philadelphia, PA: Taylor & Francis.

Wogalter, M. S. (2006). Handbook of warnings. In *Handbook of warnings* (pp. xxi, 841). Mahwah, NJ: Lawrence Erlbaum Associates Publishers.

**2) Articles Excluded: participants were identified as being other than the public (e.g., health care professionals)**

Abraham, T. (2009). Risk and outbreak communication: Lessons from alternative paradigms. *Bulletin of the World Health Organization*, *87*, 604-607.

Arya, D. & Nicholls, D. (2005). Identifying and communicating clinical risk. *Australasian Psychiatry*, *13*, 366-370.

Bomlitz, L. J. & Brezis, M. (2008). Misrepresentation of health risks by mass media. *Journal of Public Health* (Oxford, England), *30*, 202-204.

Breakwell, G. M. (2000). Risk communication: Factors affecting impact. *British Medical Bulletin*, *56*, 110-120.

Briggs, D. & Stern, R. (2007). Risk responses to environmental hazards to health – Towards an ecological approach. *Journal of Risk Research*, *10*, 593-622.

Broz, D., Levin, E. C., Mucha, A. P., Pelzel, D., Wong, W., Persky, V. W. et al. (2009). Lessons learned from Chicago's emergency response to mass evacuations caused by Hurricane Katrina. *American Journal of Public Health*, *99*, 1496-1504.

Buehler, J. W., Whitney, E. A., & Berkelman, R. L. (2006). Business and public health collaboration for emergency preparedness in Georgia: A case study. *BMC Public Health*, *6,* 1-13.

Butts, C. T. (1998). *Generating panic within populations.* Department of Social and Decision Sciences and Centre for the Computational Analysis of Social Organizational Systems,i-32.

Butts, C. T., Petrescu-Prahova, M., & Cross, B. R. (2007). Responder communication networks in the World Trade Center disaster: Implications for modeling of communication within emergency settings. *The Journal of Mathematical Sociology*, *31*, 121-147.

Chen, K., Chen, W. J., Malilay, J., & Twu, S. (2003). The public health response to the Chi-Chi earthquake in Taiwan, 1999. *Public Health Reports*, *118*, 493-499.

Chess, C., Salomone, K. L., & Hance, B. J. (1995). Improving risk communication in government: Research priorities. *Risk Analysis*, *15*, 127-135.

Chess, C., Reilly, M. A., & Cuite, C. (2009). Government as biosecurity communicator: The 2006 spinach advisory. *Biosecurity and Bioterrorism: Biodefense Strategy, Practice, and Science*, *7*, 144-152.

Collins, J. J., Bodner, K. M., Baase, C. M., Burns, C., Jammer, B., & Bloemen, L. J. (2004). Communication of epidemiology study results by industry: The Dow Chemical Company approach. *Journal of Exposure Analysis and Environmental Epidemiology*, *14*, 492-497.

Covello, V. T. (2003). Best practices in public health risk and crisis communication. *Journal of Health Communication*, *8* (Suppl 1), 5-8.

Dolores, J. C. (2008). *The impact of differential forms of risk communication on judicial decision-making*. Dolores, John Christian: Drexel U, USEditor.

Feldman, D. L. (1993). SARA title III and community hazards planning: The case of the chemical stockpile emergency preparedness program. *International Journal of Mass Emergencies and Disasters*, *11*, 85-97.

Fisher, A., Chitose, A., & Gipson, P. S. (1994). One agency's use of risk assessment and risk communication. *Risk Analysis*, *14*, 207-212.

Freudenburg, W. R. & Rursch, J. A. (1994). The risks of "Putting the numbers in context": A cautionary tale. *Risk Analysis*, *14*, 949-958.

Funk, S., Gilad, E., Watkins, C., & Jansen, V. A. A. (2009). The spread of awareness and its impact on epidemic outbreaks. *Proceedings of the National Academy of Sciences of the United States of America*, *106*, 6872-6877.

Galvez, M. P., Peters, R., Graber, N., & Forman, J. (2007). Effective risk communication in children's environmental health: Lessons learned from 9/11. *Pediatric Clinics of North America*, *54*, 33-46.

Goddard, N. L., Delpech, V. C., Watson, J. M., Regan, M., & Nicoll, A. (2006). Lessons learned from SARS: The experience of the health protection ageny, England. *Public Health*, *120*, 27-32.

Kapucu, N., Berman, E. M., & Wang, X. (2008). Emergency information management and public disaster preparedness: Lessons from the 2004 Florida hurricane season. *International Journal of Mass Emergencies and Disasters*, *26*, 169-196.

Kilijanek, T. S., Drabek, T. E., Adams, C. R., & Tamminga, H. L. (1979). The emergence of a post-disaster communication network. Unpublished work.

Lofstedt, R. (2003). Risk communication: Pitfalls and promises. *European Review*, *11*, 417-435.

Lowrey, W., Evans, W., Gower, K. K., Robinson, J. A., Ginter, P. M., McCormick, L. C., et al. (2007). Effective media communication of disasters: Pressing problems and recommendations. *BMC Public Health*, *7*, 97.

Menon, K. U. (2006). SARS revisited: Managing "outbreaks" with "communications." *Annals of the Academy of Medicine, Singapore*, *35*, 361-367.

Neuwirth, K., Dunwoody, S., & Griffin, R. J. (2000). Protection motivation and risk communication. *Risk Analysis*, *20*, 721-734.

Potter, M. A., Sweeney, P., Thomas, C., Miller, T. M., & Gourley, T. (2005). Connecting silos: The legal bases for public health emergency response in Pennsylvania. *Journal of Public Health Management and Practice*, (*11)*. S50-56.

Rogers, W. A., Street, J. M., Braunack-Mayer, A. J., & Hiller, J. E. (2009). Pandemic influenza communication: Views from a deliberative forum. *Health Expectations: An international journal of public participation in health*, *12*, 331-342.

Rundblad, G. (2008). The semantics and pragmatics of water notices and the impact on public health. *Journal of Water and Health*, *6*, 77-86.

Sandman, P. M. (2005). Responding to community outrage: Strategies for effective risk communication. *Journal of Environmental Health*, *67*, 30.

Skanavis, C., Koumouris, G. A., & Petreniti, V. (2005). Public participation mechanisms in environmental disasters. *Environmental Management*, *35*, 821-837.

Sly, T. (2000). The perception and communication of risk: A guide for the local health agency. *Canadian Journal of Public Health*, *91*, 153-155.

Smith, R. D. (2006). Responding to global infectious disease outbreaks: Lessons from SARS on the role of risk perception, communication and management. *Social Science and Medicine*, *63*, 3113-3123.

Springston, J. K. & Lariscy, R. A. W. (2005). Public relations effectiveness in public health institutions. *Journal of Health and Human Services Administration*, *28*, 218-245.

Thomas, C. W., Vanderford, M. L., & Quinn, S. C. (2008). Evaluating emergency risk communications: A dialogue with the experts. *Health Promotion Practice*, *(9)*, S5-12

Ting, H. C. & Wallsten, T. S. (2008). Learning to communicate risk information in groups. *Judgment and Decision Making*, *3*, 659-666.

Tinker, T. L. (1996). Recommendations to improve health risk communication: Lessons learned from the U.S. Public Health Service. *Journal of Health Communication*, *1*, 197-217.

Tinker, T. L., Collins, C. M., King, H. S., & Hoover, M. D. (2000). Assessing risk communication effectiveness: Perspectives of agency practitioners. *Journal of Hazardous Materials*, *73*, 117-127.

Valenti, J. M., Ackland, L., & Steele, K. D. (1998). Nuclear waste, secrecy and the mass media. *Science and Engineering Ethics*, *4*, 181-190.

van den Burg, S. W. K. & Mol, A. P. J. (2008). Making it all publicly available: Four challenges to environmental disclosure. In M. Bostrom & C. Garsten (Eds.), *Organizing transnational accountability* (pp. 177-193). Northampton, MA: Edward Elgar Publishing.

Verschuur, M., Spinhoven, P., van Emmerik, A., & Rosendaal, F. (1430). Making a bad thing worse: Effects of communication of results of an epidemiological study after an aviation disaster. *Social Science and Medicine*, *65*, 1430-1441.

**3) Articles Excluded: Intervention(s) were not Community-Based**

Abrahamson, V., Wolf, J., Lorenzoni, I., Fenn, B., Kovats, S., Wilkinson, P., et al. (2009). Perceptions of heatwave risks to health: Interview-based study of older people in London and Norwich, UK. *Journal of Public Health* (Oxford, England), *31*, 119-126.

Adeyanju, C. T. (2005). Discourse of health risks and anti-racial diversity: An analysis of media coverage of the non-ebola panic in Hamilton. *Dissertation Abstracts International, A: The Humanities and Social Sciences*, *66*, 2399-239A.

Burger, J., Gochfeld, M., & Pletnikoff, K. (2009). Collaboration versus communication: The Department of Energy's Amchitka Island and the Aleut community. *Environmental Research*, *109*, 503-510.

Campbell, V. A., Gilyard, J. A., Sinclair, L., Sternberg, T., & Kailes, J. I. (2009). Preparing for and responding to pandemic influenza: Implications for people with disabilities. *American Journal of Public Health*, *99*, S294-S300.

Chipman, H., Kendall, P., Slater, M., & Auld, G. (1996). Audience responses to a risk communication message in four media formats. *Journal of Nutrition Education*, *28*, 133-139.

Cutchin, M. P., Martin, K. R., Owen, S. V., & Goodwin, J. S. (2008). Concern about petrochemical health risk before and after a refinery explosion. *Risk Analysis*, *28*, 589-601.

Elledge, B. L., Brand, M., Regens, J. L., & Boatright, D. T. (2008). Implications of public understanding of avian influenza for fostering effective risk communication. *Health Promotion Practice*, (*9)*, S54-59

Fessenden-Raden, J., Fitchen, J. M., & Heath, J. S. (1987). Providing risk information in communities: Factors influencing what is heard and accepted. *Science, Technology, and Human Values*, *12*, 3-4.

Fisher, A. & Chen, Y. C. (1996). Customer perceptions of agency risk communication. *Risk Analysis*, *16*, 177-184.

Folmer, C. P. R. & Van Lange, P. A. M. (1016). Why promises and threats need each other. *European Journal of Social Psychology*, *37*, 1016-1031.

Freimuth, V. S., Hilyard, K. M., Barge, J. K., & Sokler, L. A. (2008). Action, not talk: A simulation of risk communication during the first hours of a pandemic. *Health Promotion Practice*, (*9),* S35-44.

Frewer, L. J., Howard, C., Hedderley, D., & Shepherd, R. (1997). The elaboration likelihood model and communication about food risks. In *Risk analysis* *(17),* 759-770.

Hagemeier-Klose, M. & Wagner, K. (2009). Evaluation of flood hazard maps in print and web mapping services as information tools in flood risk communication. *Natural Hazards and Earth System Sciences*, *9*, 563-574.

Heath, R. L. & Palenchar, M. (2000). Community relations and risk communication: A longitudinal study of the impact of emergency response messages. *Journal of Public Relations Research*, *12*, 131-161.

Johnson, B. B. (2003). Are some risk comparisons more effective under conflict? A replication and extension of Roth et al. *Risk Analysis*, *23*, 767-780.

Johnson, B. B. (2004). Risk comparisons, conflict, and risk acceptability claims. *Risk Analysis*, *24*, 131-145.

Kapucu, N. (2008). Collaborative emergency management: Better community organising, better public preparedness and response. *Disasters*, *32*, 239-262.

Kornelis, M., de Jonge, J., Frewer, L., & Dagevos, H. (2007). Consumer selection of food-safety information sources. *Risk Analysis*, *27*, 327-335.

Lave, T. R. & Lave, L. B. (1991). Public perception of the risks of floods: Implications for communication. *Risk Analysis*, *11*, 255-267.

Nerb, J. & Spada, H. (2001). Evaluation of environmental problems: A coherence model of cognition and emotion. *Cognition and Emotion*, *15*, 521-551.

Nicholas, D. B., Gearing, R. E., Koller, D., Salter, R., & Selkirk, E. K. (2008). Pediatric epidemic crisis: Lessons for policy and practice development. *Health Policy*, *88*, 2-3.

Palmer, S., Brown, D., & Morgan, D. (1256). Early qualitative risk assessment of the emerging zoonotic potential of animal diseases. *British Medical Journal*, *331*, 1256-1260.

Payne-Sturges, D. C., Schwab, M., & Buckley, T. J. (2004). Closing the research loop: A risk-based approach for communicating results of air pollution exposure studies. *Environmental Health Perspectives*, *112*, 28-34.

Quah, S. R. & Hin-Peng, L. (2004). Crisis prevention and management during SARS outbreak, Singapore. *Emerging Infectious Diseases*, *10*, 364-368.

Rebmann, T., Carrico, R., & English, J. F. (2008). Lessons public health professionals learned from past disasters. *Public Health Nursing*, *25*, 344-352.

Slovic, P., Malmfors, T., Krewski, D., Mertz, C. K., Neil, N., & Bartlett, S. (1995). Intuitive toxicology. II. Expert and lay judgments of chemical risks in Canada. *Risk Analysis*, (*15)*, 661-675.

Spence, P. R., Lachlan, K., Burke, J. M., & Seeger, M. W. (2009). Media use and information needs of the disabled during a natural disaster. In V. M. Brennan (Ed.), *Natural disasters and public health: Hurricanes Katrina, Rita, and Wilma* (pp. 180-190). Baltimore, MD: Johns Hopkins University Press; US.

ter Huurne, E. F. J., Griffin, R. J., & Gutteling, J. M. (2009). Risk information seeking among US and Dutch residents: An application of the model of risk information seeking and processing. *Science Communication*, *31*, 215-237.

Visschers, V. H. M., Meertens, R. M., Passchier, W. F., & deVries, N. K. (2007). How does the general public evaluate risk information? The impact of associations with other risks. *Risk Analysis*, *27*, 715-727.

Wray, R. J., Becker, S. M., Henderson, N., Glik, D., Jupka, K., Middleton, S., et al. (2008). Communicating with the public about emerging health threats: Lessons from the Pre-Event Message Development Project. *American Journal of Public Health*, *98*, 2214-2222.

**4) Articles Excluded: Outcomes of Interest were not Reported**

Frewer, L. J. & Miles, S. (2003). Temporal stability of the psychological determinants of trust: Implications for communication about food risks. *Health, Risk & Society, 5,* 259-271.

Golding, D., Krimsky, S., & Plough, A. (1992). Evaluating risk communication: Narrative vs. technical presentations of information about radon. *Risk Analysis, 12,* 27-35.

Heath, R. L., Bradshaw, J., & Lee, J. (2002). Community relationship building: Local leadership in the risk communication infrastructure. *Journal of Public Relations Research, 14,* 317-353.

Maffei, C., Stanislao, F. D., & Renga, G. (1990). Epidemics and the media: An Italian experience. *Public Health, 104,* 33-35.

Parrott, R., Hopfer, S., Ghetian, C., & Lengerich, E. (2007). Mapping as a visual health communication tool: Promises and dilemmas. *Health Communication, 22,* 13-24.
