# Supplementary material for: Communication about environmental health risks: A systematic review
Source: Environ Health. 2010 Nov 1;9:67. doi: 10.1186/1476-069X-9-67 (PMC2988771; doi:10.1186/1476-069X-9-67)
Supplement: Additional file 5 — Quality Assessment Tool for Qualitative Studies. This file contains the tool used to assess the methodological quality of qualitative primary studies that passed initial relevance screening for inclusion in this review. [file 1476-069X-9-67-S5.DOC]

**Additional File 5. Quality Assessment Tool for Qualitative Studies**

| **CITATION:** | |
| --- | --- |
|  | **Comments** |
| **STUDY PURPOSE:**  Was the purpose and/or research  question stated clearly?  􀁻 yes  􀁻 no | Outline the purpose of the study and/or research question. |
| **LITERATURE:**  Was relevant background  literature reviewed?  􀁻 yes  􀁻 no | Describe the justification of the need for this study. Was it clear and compelling? |
|  | How does the study apply to your practice and/or to your research question? Is it worth  continuing this review?1 |
| **STUDY DESIGN:**  What was the design?  􀁻 phenomenology  􀁻 ethnography  􀁻 grounded theory  􀁻 participatory action research  􀁻 other  ______________________ | Was the design appropriate for the study question? (i.e., rationale) Explain. |
| Was a theoretical perspective  identified?  􀁻 yes  􀁻 no | Describe the theoretical or philosophical perspective for this study e.g., researcher’s  perspective. |
| Method(s) used:  􀁻 participant observation  􀁻 interviews  􀁻 document review  􀁻 focus groups  􀁻 other  ______________________ | Describe the method(s) used to answer the research question. Are the methods congruent with  the philosophical underpinnings and purpose? |
| **SAMPLING:**  Was the process of purposeful  selection described?  􀁻 yes  􀁻 no | Describe sampling methods used. Was the sampling method appropriate to the study purpose  or research question? |
| Was sampling done until  redundancy in data was reached?2  􀁻 yes  􀁻 no  􀁻 not addressed | Are the participants described in adequate detail? How is the sample applicable to your  practice or research question? Is it worth continuing? |
| Was informed consent obtained?  􀁻 yes  􀁻 no  􀁻 not addressed |  |
| **DATA COLLECTION:**  **Descriptive Clarity**  Clear & complete description of  site: 􀁻 yes 􀁻 no  participants: 􀁻 yes 􀁻 no  Role of researcher & relationship  with participants:  􀁻 yes 􀁻 no  Identification of assumptions and  biases of researcher:  􀁻 yes 􀁻 no | Describe the context of the study. Was it sufficient for understanding of the “whole” picture?  What was missing and how does that influence your understanding of the research? |
| **Procedural Rigour**  Procedural rigor was used in data  collection strategies?  􀁻 yes  􀁻 no  􀁻 not addressed | Do the researchers provide adequate information about data collection procedures e.g.,  gaining access to the site, field notes, training data gatherers? Describe any flexibility in the  design & data collection methods. |
| **DATA ANALYSES:**  **Analytical Rigour**  Data analyses were inductive?  􀁻 yes 􀁻 no 􀁻 not addressed  Findings were consistent with &  reflective of data?  􀁻 yes 􀁻 no | Describe method(s) of data analysis. Were the methods appropriate? What were the findings? |
| **Auditability**  Decision trail developed?  􀁻 yes 􀁻 no 􀁻 not addressed  Process of analyzing the data was  described adequately?  􀁻 yes 􀁻 no 􀁻 not addressed | Describe the decisions of the researcher re: transformation of data to codes/themes. Outline  the rationale given for development of themes. |
| **Theoretical Connections**  Did a meaningful picture of the  phenomenon under study emerge?  􀁻 yes  􀁻 no | How were concepts under study clarified & refined, and relationships made clear? Describe  any conceptual frameworks that emerged. |
| **OVERALL RIGOUR**  Was there evidence of the four  components of trustworthiness?  Credibility 􀁻 yes 􀁻 no  Transferability 􀁻 yes 􀁻 no  Dependability 􀁻 yes 􀁻 no  Comfirmability 􀁻 yes 􀁻 no | For each of the components of trustworthiness, identify what the researcher used to ensure  each.  What meaning and relevance does this study have for your practice or research question? |
| **CONCLUSIONS &**  **IMPLICATIONS**  Conclusions were appropriate  given the study findings?  􀁻 yes 􀁻 no  The findings contributed to theory  development & future OT  practice/ research?  􀁻 yes 􀁻 no | What did the study conclude? What were the implications of the findings for occupational  therapy (practice & research)? What were the main limitations in the study? |
| © **Letts, L., Wilkins, S., Law, M., Stewart, D., Bosch, J., & Westmorland, M., 2007 McMaster University** | |
| 1 When doing critical reviews, there are strategic points in the process at which you may decide the research is not applicable to your practice and question. You may decide then that it is not worthwhile to continue with the review.  2 Throughout the form, “no” means the authors explicitly state reasons for not doing it; “not addressed” should be ticked if there is no mention of the issue. | |
