# Supplementary material for: Communication about environmental health risks: A systematic review
Source: Environ Health. 2010 Nov 1;9:67. doi: 10.1186/1476-069X-9-67 (PMC2988771; doi:10.1186/1476-069X-9-67)
Supplement: Additional file 6 — Data Extraction Results for Included Quantitative Studies. This file contains the data extraction results for all 24 quantitative primary articles included in this review that were assessed for methodological quality. Data included authors, date of publication, objective, methods, participants, interventions, measurement instrument, and outcomes. [file 1476-069X-9-67-S6.DOC]

**Additional File 6. Data Extraction Results for Included Quantitative Studies (n = 21)**

**[in alphabetical order]**

| **Study** | | Angulo et al. (1997) |
| --- | --- | --- |
| **Objective** | | To test the effectiveness of a boil water order during a *Salmonella typhimurium* outbreak. |
| **Methods** | | *Design:* one-time telephone interviews (surveys)  *Selection:* random sample of 150 households from the community tax roster  *Blinding:* not applicable |
| **Participants** | | *Sample:* (*N* = 120) households with 329 members  *Characteristics:* household; the age and sex distributions of the participants were similar to 1990 U.S. census figures  *Withdrawals/Drop-outs:* not applicable |
| **Intervention** | | The researchers assessed the effectiveness of a boil water order. |
| **Measurement Instrument:** | | Survey |
| **Outcomes** | | Many residents (31%) drank unboiled water after being informed about  the order.  12 of 14 people who developed diarrhea after the issuance of the boil water order reported drinking unboiled water after being informed about the order.  The most common reasons were forgetting (44%), not believing the initial notification (25%) and not understanding that ice should be made with boiled water (17%).  Levels of statistical significance were not reported. |
| **Comments** | | The initial boil water order did not explain the reason for the issuance and did not mention associated illness. |
| **Study** | Atlas (2007) | |
| **Objective** | To investigate public knowledge and use of information for the U.S. Toxics Release Inventory (TRI). | |
| **Methods** | *Design:* three-phase survey  *Selection:* participants were selected through random-digit dialling in  two U.S. counties  *Blinding:* not applicable | |
| **Participants** | *Sample:* (*N* = 1292); Wake County (*n* = 646); Baltimore County (*n* = 646)  *Characteristics:* the demographics of the survey respondents were compared to the 2000 Census data of their respective counties and the U.S. as a whole; there were differences in ethnicity, education and income between the represented counties  *Withdrawals/Drop-outs:*  Phase 2 – Wake County (*n* = 504) 78%; Baltimore County (*n* = 470) 73%.  Phase 3 – Wake County (*n* = 448) 69%; Baltimore County (*n* = 399) 62%. | |
| **Intervention** | No specific intervention was used, but rather a measurement was made of the public’s awareness of an existing program. | |
| **Measurement Instrument:** | Phase 1 of the survey measured baseline knowledge of TRI information; Phase 2 measured whether the publication of 1999 of TRI data affected respondents’ knowledge of TRI; in Phase 3, participants were re-interviewed after being sent information about the 1999 data plus a website address where they could obtain additional TRI information. | |
| **Outcomes** | Respondents had a low recall and recognition of the TRI-related information.  Levels of statistical significance were not reported. | |

| **Study** | Blendon et al. (2007) |
| --- | --- |
| **Objective** | To determine the reasons why some of the people threatened by Hurricanes Katrina and Rita did not evacuate. |
| **Methods** | *Design:* one-time survey  *Selection:* random digit dialling with an adult (≥18 years) respondent for each household being randomly selected  *Blinding:* not reported |
| **Participants** | *Sample:* (*N* = 2,006); East Baton Rouge Parish (*n* = 500), Harris County Texas (*n* = 505), Mississippi and Alabama excluding but adjacent to counties near the Gulf that had been declared disaster areas (*n* = 501), Dallas County Texas (*n* = 500); Dallas was used as a comparison area because although it was not damaged by the hurricanes, it was originally expected to be hit by Hurricane Rita; the other three counties were chosen because they were heavily impacted by one or both of the hurricanes but were outside the main area of heavy damage where telephone communication was not possible  *Characteristics:* adults  *Withdrawals/Drop-outs:* (*n* = 1116) adults who completed the interviews – 56% |
| **Intervention** | In hypothetical situations about a natural disaster such as a hurricane or flood, the researchers asked respondents if they would evacuate if told to do so by a government official and if not, why they would not leave. |
| **Measurement Instrument:** | Interview |
| **Outcomes** | Reasons people did not leave: thought they would be safe at home (73–79%); thought that the hurricane and its aftermath would not be too bad (42–51%); need to protect property (20–31%); not able to get gas (16–29%); did not know where to go to be safe (11–21%); could not afford to leave (8–23%); tried but unable to leave (6–21%); did not want to leave pets (10–22%); physically could not leave (5–11%); or, caring for someone who could not leave (8–16%).  Most said they would be very or somewhat interested in learning more about outside sources to be prepared (57–66%) and how to evacuate if there was another major hurricane (61–69%).  Source of information: approximately half of the respondents said they would be very or somewhat likely to contact their local health department (52–56%); local emergency services (49–59%); state health department (48–57%); and the Center for Disease Control and Prevention (45–50%).  Respondents in Houston were more likely (*p* < .05) to evacuate than were residents of Baton Rouge or Mississippi/Alabama. |
| **Comments** | African Americans and Latinos were oversampled so that those sample sizes would be adequate for statistical analysis. |

| **Study** | Bord & O'Connor (1990) |
| --- | --- |
| **Objective** | To explore reactions to food irradiation. |
| **Methods** | *Design:* controlled clinical trial  *Selection:* women’s business, social, civic or religious groups were contacted and informed about the research project; interested groups were contacted and a meeting place and time were scheduled  *Blinding:* not reported |
| **Participants** | *Sample:* (*N* = 195)  *Characteristics:* the sample consisted of adult women across Pennsylvania  *Withdrawals/Drop-outs:* not reported |
| **Intervention** | Participants were screened to determine baseline attitudes and then given one of eight structured communications about irradiated foods. The communications included an explanation of the irradiation process, with diagrams, which had a technical language and a non-technical variation. Half of the sample also received a detailed presentation of the major arguments for and against food irradiation, and half the sample received a short history of the use of irradiation food. |
| **Measurement Instrument:** | Data were gathered in a four-step process:   1. respondents completed a baseline survey 2. respondents completed one of eight structured communications about irradiated food 3. respondents completed a second survey 4. focus groups were held to probe attitudes and intentions |
| **Outcomes** | Whether respondents received a technical or non-technical communication about the food irradiation process and whether they received a detailed discussion of the major arguments for and against food irradiation had no discernible effect on their judgment. Those who had accurate information about food irradiation showed increased acceptance. Those who scored high in knowledge of food irradiation also had higher levels of education  (Pearson’s *r* = .26)*,* less distrust (*r* = –.28), lower alienation (*r* = –.20), lower anti-tech scores (*r* = –.23) and less fear of radiation (*r* =–.21)*.* |

| **Study** | Burger et al. (2003) |
| --- | --- |
| **Objective** | To examine the efficacy of presenting information about the risks of consuming contaminated fish and shellfish in two different formats: a brochure and a classroom presentation. |
| **Methods** | *Design:* cohort analytic  *Selection:* all women at a women’s health clinic in Elizabeth, New Jersey were approached and asked to participant  *Blinding:*  not reported |
| **Participants** | *Sample:* (*N* = 97); brochure (*n* = 46); classroom lesson (*n* = 51)  *Characteristics:* pregnant women with a mean age of 28 years and women of child-bearing age with a mean age of 29 years  *Withdrawals/Drop-outs:* not applicable |
| **Intervention** | Women were presented with information about the hazards of eating unsafe fish in a classroom or fact sheet format. The information presented was the same for both but the classroom lesson was longer and each idea was presented in more detail than the brochure. |
| **Measurement Instrument:** | One-on-one interviews offered in English or Spanish  Measurement instruments (brochure or classroom) were presented on a random basis |
| **Outcomes** | 96% of the women who heard the classroom presentations reported an understanding that it was unsafe to eat fish from the contaminated bay, compared with 72% of the women who received the information in a brochure.  Those who heard the lesson provided the correct answers more often than did those who read the same information in the brochure for 18 of 20 questions (*p* < .01). |
| **Comments** | These interventions were delivered to targeted women’s health clinics. Hand-delivered brochures or classroom lessons presented in a woman’s first language seem to be effective means of delivering these health warnings. |
| **Study** | Burger & Waishwell (2001) |
| **Objective** | To gain insight into anglers’ perceptions about a supplemental fact sheet consumption advisory for fish. The researchers were interested in:   1. whether participants had previously read the Fish Fact Sheet or had heard about the consumption advisories 2. what major message they obtained from the sheet 3. who they felt the fact sheet was aimed at, and who should get the fact sheet 4. who should be concerned about health risks from consuming the fish 5. the best methods of disseminating they type of information |
| **Methods** | *Design:* one-time interview  *Selection:* all fishers along Savannah River were approached  *Blinding:* not reported |
| **Participants** | *Sample:* (*N* = 92)  *Characteristics:* African-American (37%), Caucasian (62%), male (88%) with an age range of 23 to 77 years  *Withdrawals/Drop-outs:* not applicable |
| **Intervention** | Participants were given a Fish Fact Sheet, asked to read it and respond to a set of questions about the sheet. |
| **Measurement Instrument:** | Survey questionnaire developed by the research team |
| **Outcomes** | *Target Audience:* 57% indicated that everyone should get the Fact Sheet and 37% indicated that it should go to those whom it concerned (fishers, purchasers, those living by the contaminated river).  *Who should limit fish consumption:* Most (African American 86%, Caucasian 81%) indicated they felt that fish consumption should be limited by some people.  *Ways to reduce risk:* 71% of respondents indicated they felt there were ways to reduce the risk associated with contaminated fish, primarily through the reduction of consumption of contaminated fish.  *Additional desirable information:*  how to get additional copies of fact sheets and more information on the levels of contamination in fish and risk levels and ecological pathways. |
| **Comments** | Survey questions were included in the study report. Personal interaction with participants appears to increase understanding of fact sheet information. |

| **Study** | Burnside et al. (2007) |
| --- | --- |
| **Objective** | To examine the role of information and risk perception on shaping hypothetical evacuation behaviour. |
| **Methods** | *Design:* one-time survey  *Selection:* random digit dialling  *Blinding:* not reported |
| **Participants** | *Sample:* (*N* = 1,207)  *Characteristics:* households in the greater New Orleans area  *Withdrawals/Drop-outs:* not applicable |
| **Intervention** | Using a hypothetical evacuation order, respondents were asked, if public officials recommended an evacuation because of threat of a hurricane this year, what would you most likely do: definitely evacuate, probably evacuate, probably not evacuate or definitely not evacuate.  These four categories were combined into two: people who would evacuate and people who would not evacuate.  The researchers also hypothesized that people who view more visual images of hurricane damage would be more likely to evacuate. |
| **Measurement Instrument:** | Telephone survey |
| **Outcomes** | People who view public officials’ advice as an important source of information were more likely to evacuate (*p* < .001). People who viewed more visual images of hurricane damage were more likely to evacuate (*p* < .01). |

| **Study** | Connelly & Knuth (1998) |
| --- | --- |
| **Objective** | To examine four elements of risk information presentation format using printed materials on the subject of the hazards of eating contaminated fish from the Great Lakes. |
| **Methods** | *Design:* one-group post-test design  *Selection:* licensed anglers from all states with shorelines on the Great Lakes  *Blinding:* not applicable |
| **Participants** | *Sample:* (*N* = 3,546)  *Characteristics:* not reported  *Withdrawals/Drop-outs:* not applicable |
| **Intervention** | Various print materials containing a warning about consumption of Great Lake non-commercial fish were used to explore peoples’ perceptions of a) reading level (grade 5 vs. grade 11); b) use of diagrams vs. text; c) use of commanding versus cajoling tone; and d) use of qualitative vs. quantitative information presented in a risk level. |
| **Measurement Instrument:** | One-time survey |
| **Outcomes** | *Reading level using two texts describing the potential effects of chemical contamination on humans using two reading levels (grade 5 and grade 11):* 68% of respondents thought that grade 11 reading level was clearest and easiest to understand. Those with less that high school education were more likely to choose the grade 5 reading level (*p* < .01) than those who had a least a high school diploma.  *Graphics versus text only describing how to clean a fish to reduce exposure to contaminants:* 58% preferred the combined diagram and text, indicating they were easier to read and understand. Households of concern (houses with women of childbearing age and anglers living in households with children under the age of 15) were more likely to choose the text/diagram combination (*p* < .01).  *Two presentation tones: commanding, authoritative tone vs. cajoling more conservative tone:* 79% of respondents felt the cajoling tone was better at providing them with the information they needed.  *Qualitative versus quantitative risk ladder with the description of risky activities; the physical placement of fish consumption on the risk ladder was the same for both ladders; the quantitative ladder described the risk level in chances out of 1000, and the qualitative ladder described risk as high, moderate or low:* 57% felt that the quantitative ladder helped them to best understand the health risks associated with eating Great Lake fish. Households of concern were more likely to choose the quantitative ladder (*p* < .05) than were other households. |

| **Study** | Fox et al. (2006) |
| --- | --- |
| **Objective** | To determine knowledge, behaviour and attitude among Kansas residents exposed to a statewide West Nile Virus health education campaign. |
| **Methods** | *Design:* multi-modal including a telephone survey of individuals (all other surveys were with groups in industry or private providers—these results will not be reported here)  *Selection:* random selection of urban and rural counties and telephone numbers  *Blinding:* assessors and participants were aware of the research question |
| **Participants** | *Sample:* (*N* = 516)  *Characteristics:* under 50 years (53%), some high school (63%), rural dwelling (56%), Caucasian (92%), female (63%)  *Withdrawals/Drop-outs:* not applicable |
| **Intervention** | The State Department of Health and Environment (with a private marketing firm) produced and disseminated a multimedia campaign to increase awareness of and educate about West Nile Virus. The messages were:   1. apply insect repellent containing DEET 2. wear long sleeves and pants during dawn and dusk 3. eliminate repositories of standing water 4. check and repair window screens |
| **Measurement Instrument:** | Survey was drafted and piloted outside the sample frame prior to full implementation  Survey offered in English and Spanish |
| **Outcomes** | Knowledge was widespread but preventative behaviours were not. Television (88%), newspapers (72%) and word-of-mouth (65%) were the most frequently cited sources of information. A small percentage (8%) of respondents cited health professionals as sources of information. Levels of statistical significance were not reported. |
| **Comments** | The survey was of individuals not households, therefore whoever answered the phone, if eligible, was surveyed. Phone surveys miss residents who do not have a phone or rely on cellphones. Few of the surveys were administered in Spanish (*N* = 17). |

| **Study** | Freimuth & Van Nevel (1993) |
| --- | --- |
| **Objective** | To evaluate a campaign to increase awareness of and information about the nature, extent and seriousness of asbestos exposure aimed at delivering information to a target group of manual labourers over the age of 50 who could not be individually identified. |
| **Methods** | *Design:* interrupted time series  *Selection:* participants were part of a probability omnibus survey; high risk target audiences could not be identified as individuals; geographic location was used as a surrogate targeting strategy; 16 high priority areas were identified and received more intensive dissemination materials  *Blinding:* not applicable |
| **Participants** | *Sample:* (*N* = 1500) at each of three waves of the survey  *Characteristics:* general population  *Withdrawals/Drop-outs:* not applicable |
| **Intervention** | Public service messages were developed to be delivered through a mass media campaign including radio, television and print. |
| **Measurement Instrument:** | Three waves of a national probability survey and monitoring of calls and letters |
| **Outcomes** | The percentage of people who believed they had been exposed to asbestos increased to 33% from 26% between the pre- and post-campaign surveys. Post-campaign level of knowledge of asbestos-associated illness risks increased to 67% from 58% pre-campaign level.  Levels of statistical significance were not reported. |
| **Comments** | The study authors identified the study design as a case study. According to the definitions we use, we identified this as an interrupted time series. |

| **Study** | Gutteling (1993) |
| --- | --- |
| **Objective** | To examine the impact of the information source and explicitness of conclusions on the effectiveness of risk communication regarding new hazardous technology. |
| **Methods** | *Design:* randomized 2 × 2 post-test  *Selection:* a random sample of inhabitants of cities in the Netherlands (drawn from the post office database of telephone users) contacted by mail to participate  *Blinding:* not reported |
| **Participants** | *Sample:* (*N* = 508); intervention group (*n* = 383), control group (*n* = 125)  *Characteristics:* Male (60%); only elementary school (24%), university or professional training (25%); younger than 29 years (17%), 30–39 years (29%), 40–49 years (24%), and older than 50 (30%)  *Withdrawals/Drop-outs:* not applicable |
| **Intervention** | Participants in the experimental group received one of two brochures. The brochures were divided by source of information and identified either the national government or a private company as the source of information. The control group received a questionnaire but no brochure. |
| **Measurement Instrument:** | 2 × 2 factorial post-test-only interview |
| **Outcomes** | The experimental group showed a significant difference compared with the control group in knowledge about the technology *F*(1, 403) = 14.96, *p* < .001; attitude about the technology *F*(1, 498) = 11.53, *p* < .001; and, assessment of benefits *F*(1, 496) = 10.98, *p* < .001. No significant differences were found between groups for attitude toward establishing a plant in the neighbourhood, assessment of risks of the technology, threat, controllability, feelings of insecurity or intentions to seek information.  Brochures from the private company aroused more fear than did the brochures from the government *F*(1, 354) +5.01, *p* < .05. No differences were found between subjects who received a brochure with or without explicit conclusions. |

| **Study** | Johnson et al. (1988) |
| --- | --- |
| **Objective** | To test the sensitivity of people’s responses to alternative presentation of the same facts about radon gas risk. |
| **Methods** | *Design:* randomized controlled trial  *Selection:* homes were selected randomly within seven areas representing major geological formations across the state (New York)  *Blinding:* not reported |
| **Participants** | *Sample:* (*N* = 2,550) homes – intervention group (*n* = 2,300), comparison group (*n* = 250)  *Characteristics:* households  *Withdrawals/Drop-outs:* not reported |
| **Intervention** | Homeowners in the sample were given three radon gas monitors for their home. The first, placed in the living room, was to be returned for analysis after two to three months; the other monitors were to be returned and analyzed after one year. After the first detector was returned, homeowners received radon gas information materials; half of those with a reading below 1 pCi/1 received an information fact sheet, while all other home owners received one of five other randomly assigned booklets. Homeowners with a radon gas reading above 1 pCi/1 were also given the EPA’s *Radon Reduction Methods: A Home Owner’s Guide.*  Information booklets options were quantitative vs. qualitative and command vs. cajole approach. All versions of the brochure contained the same information about radon gas.  The comparison group received no readings or informational materials. |
| **Measurement Instrument:** | Baseline and follow-up survey |
| **Outcomes** | Homeowners who received a single-page fact sheet did not improve their scores on the risk questions in the follow-up survey. The comparison group—without any information materials—improved their scores (might be a result of selection bias or the comparison group members paying more attention to media reports following their involvement with the baseline survey).  Older people had lower scores in the follow-up survey than they had in the baseline survey. People with more education and prior awareness of radon had better overall scores.  The command-qualitative booklet showed the greatest increase in learning about the health effects of radon. |
| **Comments** | The report authors did not provide statistical information to support their outcome claims. |

| **Study** | Major (1993) |
| --- | --- |
| **Objective** | To test Situation Communication Theory in the context of a disaster communication—specifically, an earthquake. |
| **Methods** | *Design:* interrupted time series  *Selection:* computer generated random digit dial  *Blinding:* not reported |
| **Participants** | *Sample:* first survey (*N* = 629) in Cape Girardeau-Jackson Scott City, Missouri, follow-up survey (*N* = 998) (502 adult respondents from Carbondale, Illinois and 496 adult respondents from Cape Girardeau-Jackson Scott City, Missouri  *Characteristics:* not reported  *Withdrawals/Drop-outs:* not applicable |
| **Intervention** | A series of interview questions were delivered to determine the level of involvement for the respondent in a disaster situation: problem recognition, constrained recognition, fatals or routines. |
| **Measurement Instrument:** | Baseline and follow-up survey  Problem recognition was measured by asking: “How often do you stop to think about a major earthquake hitting the area?” Answers: very often, sometimes, not often, almost never  Constraint recognition was measured by asking: “If you personally tried to do something to help protect yourself or your family from a major earthquake, do you think your efforts would make a lot of difference, some difference, not much difference or no difference at all?” |
| **Outcomes** | The constrained and problem-facers, in contrast with the fatals, reported that the earthquake problem was personally important and that they sought information and clarification about the prediction’s meaning from other people and the media.  Problem-facers and constrained were more likely to send for government booklets about earthquake safety than were the fatals.  Problem-facers found television and radio news reports helpful in preparing them for the earthquake.  Those identified as constrained talked with family members and made preparations for an earthquake.  Unlike the problem-facers, the constrained sought ways to reduce their perceived constraints by making preparations. |
| **Comments** | High problem recognition publics were more likely to have reported involvement in the earthquake issue, to have spoken with others about earthquakes and to have done something to prepare for an earthquake. |

| **Study** | Mileti & O'Brien (1992) |
| --- | --- |
| **Objective** | To test risk communication theory about public response to communicated warnings issued during an ongoing disaster (aftershocks) for the first time. |
| **Methods** | *Design:* one-time survey  *Selection:* an enumeration of residents’ names and household addresses in two U.S. counties (San Francisco and Santa Cruz) were obtained and a systematic random sample was selected from each sampling frame  *Blinding:* not reported |
| **Participants** | *Sample:* (*N* = 1652); San Francisco (*n* = 734), Santa Cruz (*n* = 918)  *Characteristics:* sample was assessed for representativeness by comparing sample characteristics with population data – sample and population data on average household size were virtually identical; females were overrepresented by 3%; ethnic groups were underrepresented in both counties by 8% and 4%; and renters were underrepresented by 23% and 12% respectively  *Withdrawals/Drop-outs:* not applicable |
| **Intervention** | The public response to aftershock warnings delivered in the media (newspaper, radio or TV) was measured. |
| **Measurement Instrument:** | A questionnaire that had been constructed, pretested, revised and mailed to respondents twice |
| **Outcomes** | **Public response to short-term (72 hours) aftershock warnings**   |  | **San Francisco** | | **Santa Cruz** | | | --- | --- | --- | --- | --- | |  | **%** | **N** | **%** | **N** | | Sought general earthquake information | 21.1 | 155 | 21.8 | 200 | | Sought information on what to do about aftershocks | 14.6 | 107 | 18.6 | 171 | | Read earthquake information in phone book | 21.9 | 161 | 13.4 | 123 | | Developed an emergency plan | 31.5 | 231 | 43.8 | 402 | | Made household items safer | 45.2 | 332 | 70.3 | 645 | | Made dwelling structurally safer | 7.4 | 054 | 17.6 | 162 |   **Public response to two-month aftershock warnings**   |  | **San Francisco** | | **Santa Cruz** | | | --- | --- | --- | --- | --- | | **%** | **N** | **%** | **N** | | Sought general earthquake information | 14.0 | 103 | 18.4 | 169 | | Sought information on what to do about aftershocks | 13.8 | 101 | 16.8 | 154 | | Read earthquake information in phone book | 17.7 | 130 | 11.3 | 104 | | Developed an emergency plan | 28.9 | 212 | 42.4 | 389 | | Made household items safer | 37.7 | 277 | 63.5 | 583 | | Made dwelling structurally safer | 12.4 | 091 | 27.2 | 250 | |
| **Comments** | The data consistently revealed that protective warning responses were more likely in Santa Cruz county, which had experienced more damage than the residents of San Francisco. Experiencing loss in a disaster may make subsequent warnings more salient, thereby enhancing the likelihood of engaging in protective actions in response to the warning. The lack of mainshock damage may have created a normalization bias for non-victims, wherein those who experience an effect during the first impact think they will avoid the damage of the aftershocks. |

| **Study** | Mulilis & Lippa (1990) |
| --- | --- |
| **Objective** | To use a persuasive message containing threatening information to test the ability of the revised protection motivation theory to predict subjects’ behaviour changes in earthquake preparedness over a five-week period. |
| **Methods** | *Design:* randomized controlled trial  *Selection:* subjects were contacted in their home and asked to participate in a study about earthquake preparedness  *Blinding:* not reported |
| **Participants** | *Sample:* (*N* = 243)  *Characteristics:*  California homeowners; the sample was compared with data from the 1980 Federal Census which showed that the sample was representative of the population  *Withdrawals/Drop-outs:* 114 completed the second questionnaire (47%) |
| **Intervention** | Manipulation Essay: Subjects read a four-paragraph information essay about earthquakes designed to manipulate the subjects’ beliefs around four dimensions: subjective probability of occurrence of a large earthquake, expected severity of damage due to a large earthquake, perceived effectiveness of earthquake preparedness, and perceived capability if earthquake preparedness. Subjects read one of 16 different essays (or 16 different combinations of the four paragraphs—one paragraph dealing with either low or high condition of each of the four dimensions). |
| **Measurement Instrument:** | *Questionnaire included:* The Mulilis-Lippa Earthquake Preparedness Scale (MLEPS); demographic and earthquake history information; experimental manipulation essay (see Intervention); and manipulation checks.  Behaviour change in earthquake preparedness was taken to be the difference between the subject’s MLEPS score on the initial and the follow-up questionnaires |
| **Outcomes** | The investigation showed that the respondents’ scores on the earthquake preparedness scale (MLEPS) increased significantly (*p* = .04) over a five week period after reading a negative, threat-inducing persuasive communication, and the behaviour change was not just a result of completing the scale. As well, the direct effect of the negative message on subjects diminished in intensity as the behaviour change increased. |

| **Study** | Natter & Berry (2005) |
| --- | --- |
| **Objective** | To compare relative and absolute forms of presenting risk information about influenza and the need for vaccination, and to investigate whether differences in people’s risk estimates and their evaluation of risk information, as a result of the different presentation formats, are still as apparent when they are provided information about the baseline level of risk. |
| **Methods** | *Design:* randomized controlled trial **–** two-factor between subject design (with/without baseline × relative/absolute risk reduction)  *Selection:* individuals were approached in a British own centre and asked to participate  *Blinding:* not reported |
| **Participants** | *Sample:* (*N* = 220), participants were randomly assigned to one of four groups, with 55 in each group  *Characteristics:* women (57%), post-secondary education (34.6%), mean age 28.2, standard deviation = 10.9, all spoke and wrote English  *Withdrawals/Drop-outs:* not applicable (one-time survey) |
| **Intervention** | Participants were provided with a fictitious scenario and a questionnaire-based survey.  Half the participants in both risk-reduction formats were informed about the baseline risk with the sentence: “It is predicted that 10% of the adult population (i.e., 10 out every 100 adults) will be affected by the flu.”  The scenario also informed participants that people were being advised to get vaccinated.  Absolute risk reduction was communicated as: “With vaccination, the risk of being affected by the flu is 5% lower.”  Relative risk reduction was communicated as: “With vaccination, the risk of being affected by the flu is reduced by 50%.” |
| **Measurement Instrument:** | Survey |
| **Outcomes** | Numerical estimates of the risk of flu without the vaccination were significantly higher (*p* < .001) if the baseline was not communicated.  Numerical estimates for the risk of flu with vaccination were significantly higher (*p* < .001) if the baseline was not communicated.  Given the absolute information, participants were more satisfied (*p* < .05) with the information than those in the relative condition, but only when informed about the baseline.  Participants given information in a relative format were more likely (*p* < .01) to indicate they would get vaccinated, but only if they were not informed of the baseline information. |

| **Study** | Predy et al. (1997) |
| --- | --- |
| **Objective** | To determine the effectiveness of a recorded information line in communicating health risk during the emergence of a new disease, hantavirus pulmonary syndrome (HPS), and to study the accuracy of recall of information about the virus among the general public. |
| **Methods** | *Design:* one-time survey  *Selection:* randomly selected telephone numbers in one Canadian city  *Blinding:* not reported |
| **Participants** | *Sample:* (*N* = 740)  *Characteristics:* adults 18 and over living in the City of Edmonton or within the extended flat rate calling areas around the city; temporary residents, people living in nursing homes and numbers not in service were not called  *Withdrawals/Drop-outs:* not applicable |
| **Intervention** | Telephone information line |
| **Measurement Instrument:** | One-time survey |
| **Outcomes** | 2% of people received their information from the recorded line and more people remembered receiving their information through the news media, particularly television at 74% and newspaper at 57%.  Of the people (3%) who called the information line, only 2% indicated that they reached the line and listened. Many reported that the line was busy when they tried to call.  30% of respondents took action to clean up mouse droppings or otherwise to prevent them from coming in contact with mice or their droppings. |
| **Comments** | The authors indicated that the media got much of their information from the information line, although there is no data to support this claim. |

| **Study** | Rich & Conn (1995) |
| --- | --- |
| **Objective** | To test the effectiveness of a computerized and automatic dialing emergency notification system in delivering pre-emergency information. |
| **Methods** | *Design:* clinical controlled trial  *Selection:* taken from single-family residents with listed or voluntarily reported telephone numbers in Contra Costa County, California  *Blinding:* not reported |
| **Participants** | *Sample:* (*N* = 351); experimental group (*n* = 209), control 1 (*n* = 74),  control 2 (*n* = 69)  *Characteristics:* male (46%, 47%, 50% respectively), under 40 years (32%, 26%, 16%), 40 to 60 years (44%, 40%, 40%), over 60 years (24%, 34%, 44%), high school or less (21%, 22%, 28%), or at least some college to graduate degree (79%, 78%, 72%)  *Withdrawals/Drop-outs:*  experimental group (*n* = 55) 26%, control 1 (*n* = 39) 53%, control 2 (*n* = 68) 99% |
| **Intervention** | A test call for emergency preparedness was issued with the option of the listener getting more information on sheltering in place.  *Intervention group:* was sent a pre-test questionnaire, received the test call and received a post-test questionnaire.  *Control Group 1:*  received the pre and post questionnaire but did not receive the test call.  *Control Group 2:* did not receive the test call and only received the post-test questionnaire. |
| **Measurement Instrument:** | Mailed questionnaires (pre- and post-test) |
| **Outcomes** | In the event of an emergency, 71% of those who received the test call knew the right agency to call compared with 11% of those who did not receive the test call.  Prior to the test call, only 20% of the 55 respondents who said they got the test call said that they had seen or heard of the instructions on how to shelter-in-place. After the call, 64% said they has seen or heard such instruction, and 77% of those said they had received the instruction through the test call.  For those who received the test call, there was an improvement in the percent of respondents who named each step in the effective sheltering (the study authors said this was statistically significant but did not provide the *p*-value).  There was a reduction in the number of respondents in the test group who indicated that they did not know what to do to shelter (from 46% to 20%), but no statistically significant change in the proportion of control group 1 who indicated that they did not know what to do.  Levels of statistical significance were not reported. |
| **Comments** | Respondents were more likely to be older, more educated and more affluent than a cross section of the public. |

| **Study** | Staats et al. (1996) |
| --- | --- |
| **Objective** | To evaluate a mass media public information campaign regarding  the effects of greenhouse gas. |
| **Methods** | *Design:* cohort (one group pre/post)  *Selection:*  not described  *Blinding:* not described |
| **Participants** | *Sample:* first survey (*N* = 965)  *Characteristics:* representative of Dutch population with respect to age and sex  *Withdrawals/Drop-outs:* follow-up survey (*n* = 704) 73% |
| **Intervention** | For a two and a half month period, 36 commercials were broadcast on national television networks and 14 advertisements appeared in national newspapers and magazines. Also during that time, billboards and posters were visible in public areas. |
| **Measurement Instrument:** | Pre- and post-survey |
| **Outcomes** | *Campaign diffusion:* 25% did not notice any of the campaign elements, 32% noticed TV-spots, posters and/or billboards, 8% only read the advertisements, 30% of the group that noticed all TV-spots, posters, billboards, and had read the advertisements, in addition to perceiving one or more of the aforementioned elements, 5% had obtained and read an information brochure.  *Knowledge of the Greenhouse Effect:* greatest increase in knowledge was with the group that had seen the television commercials and billboards and had read the advertisements (*p* < .03).  *Emotional concern:* no campaign effect on emotional concern.  *Perceived seriousness:* no campaign effect on perceived seriousness of  the problem.  *Voluntary behaviour:* only separate disposal of small chemical waste was influenced by the campaign (*p* < .0001). |
| **Comments** |  |
| **Study** | Terpstra et al. (2009) |
| **Objective** | To evaluate the effects of a small-scale communication program in the Netherlands, consisting of workshops and focus group discussions, to determine whether direct personal experience and vicarious experience obtained through risk communication cause changes to individuals’ beliefs and attitudes toward flood risk. |
| **Methods** | *Design:* cohort analytic  *Selection:* sample (*N* = 80) was drawn from a group of candidates who ran unsuccessfully in previous elections to be the general administrator of the local water board (Friesland), a number of social and community platforms (Flevoland) and from a database (Groningen)  *Blinding:* cannot tell |
| **Participants** | *Sample:* (*N* = 80)*;* workshop (*n* = 24), focus group discussions (*n* = 16), control group (*n* = 40)  *Characteristics:* only gender and age were gathered; females (44%), age range 20–70 years  *Withdrawals/Drop-outs:* not reported |
| **Intervention** | Workshop participants had a multisession experiential workshop with experts on flood risk.  Focus group participants spent time discussing flood risk but without experts.  Control group received no information |
| **Measurement Instrument:** | One-time questionnaire that had been pre-tested with a separate sample of respondents – adjustments were made  *Seven perceptions measured:* increasing risk, dread of risk, people who believe they know the risk, known to science/experts, personal control, trust of authorities and support for risk reducing measures by the public |
| **Outcomes** | There was no statistically significant difference among the three information conditions in the pre-test scores. The workshop produced statistically significant changes from the pre-test to the post-test on only two of seven dimensions (decrease in perceived societal support (*p* ≤ .05) and increase in perceptions of personal control (*p* ≤ .01)). This group also showed stable change for increasing risk, dread risk and trust (*p* ≤ .01).  Focus group showed lower levels of attitude polarization than expected.  Control group showed no significant degree of attitude polarization. |

| **Study** | Van Eijndhoven et al. (1994) |
| --- | --- |
| **Objective** | To evaluate a risk communication plan and communication plan around industrial hazardous chemical spills alerts. |
| **Methods** | *Design:* controlled clinical trial  *Selection:* information was mailed to all residents of two cities (Elst and Dordrecht) within a selected geographic area around the site of the controversial industries  *Blinding:* not reported |
| **Participants** | *Sample:* pre-intervention (*N* = 167), post-intervention (*n* = 159)  *Characteristics:* similar to the general population in the Netherlands  *Withdrawals/Drop-outs:* 6 month follow-up (*n* = 73) 48% |
| **Intervention** | Residents were mailed a two-page letter signed by the mayor and the managing director of the plant and a card with detailed instructions of what to do in case of an emergency. In addition, secondary information was available upon request. A public meeting was held in one community for those who had expressed an interest by selecting a check box on the card or letter. The purpose of the meeting was for representatives from the plant and the municipality to answer questions and concerns. |
| **Measurement Instrument:** | One-on-one interviews with either the male or female main inhabitant of the selected addresses at pre- and post-intervention times |
| **Outcomes** | Residents had a good general level knowledge of the risks posed by the plants and the potential impact of a chemical spill. The campaign had only a slight effect on that knowledge.  The initial measures indicated that the populations had little information about the emergency scenario. Only 17% knew the correct meaning of the siren signal. Following the campaign, 76% of the participants knew what the siren signal meant. But at the six-month follow-up, the campaign effect had decreased to 44%.  Levels of statistical significance were not reported. |
