# Supplementary material for: Communication about environmental health risks: A systematic review
Source: Environ Health. 2010 Nov 1;9:67. doi: 10.1186/1476-069X-9-67 (PMC2988771; doi:10.1186/1476-069X-9-67)
Supplement: Additional file 7 — Data Extraction Results for Included Qualitative Studies. This file contains the data extraction results for all 3 qualitative primary articles included in this review that were assessed for methodological quality. Data included authors, date of publication, objective, methods, participants, interventions, measurement instrument, and outcomes. [file 1476-069X-9-67-S7.DOC]

**Additional File 7. Data Extraction Results for Included Qualitative Studies (n = 3)**

**[in alphabetical order]**

| **Study** | Eisenman et al. (2007) |
| --- | --- |
| **Objective** | To better understand factors influencing evacuation decisions in impoverished communities that were affected by Hurricane Katrina. |
| **Methods** | *Design:* qualitative descriptive  *Selection:* participants were randomly selected from the three shelters that were set up to house victims of Hurricane Katrina |
| **Participants** | *Sample:* (*N* = 58)  *Characteristics:* participants who were 18 years of age or older and who were living in Louisiana before Hurricane Katrina; the participant group was predominantly African-American; 93% of participants had not evacuated until after Katrina had made landfall; the sample was older, less educated, of lower socioeconomic status and more African-American than the general population in New Orleans |
| **Intervention** | Researchers interviewed participants to determine the effect that risk communication information had on the decision to stay in the hurricane zone or to evacuate. Researchers wanted an in-depth look at what factors influenced the decision to stay or leave. |
| **Measurement Instrument:** | Semi-structured interviews |
| **Outcomes** | *Instrumental reasons:* many participants said that despite the warnings from health authorities regarding the risks of staying in the hurricane zone, they did not have the physical resources to leave (e.g., a car, a place to stay).  *Cognitive reasons:* several participants noted a lack of understanding of risk communication messages from health authorities; some participants mentioned the lack of cohesion between messages from different authorities, some telling them to stay and some telling them to leave. Many participants reported that they remember that there were televised risk warnings, but they could not recall the details of the evacuation orders.  *Risk perception:* many participants said that they did not think that the storm was going to be big or cause as much damage as it did; they thought it would be similar to other hurricanes they had experienced.  *Sociocultural reasons:* some participants felt that risks were communicated in an intentional way to protect affluent neighbourhoods without any regard for marginalized neighbourhoods. |

| **Study** | Blanchard et al. (2005) |
| --- | --- |
| **Objective** | To generate suggestions for improvement to communication in response to bioterrorism. |
| **Methods** | *Design:* qualitative descriptive  *Selection:* a convenience sample of participants recruited from flyers distributed at labour union meetings and posted at local facilities and through a local support group |
| **Participants** | *Sample:*  (*N* = 43); postal workers (*n* = 36), senate workers (*n* = 7)  *Characteristics:*  postal workers who worked during the anthrax attack – 19% were hearing impaired, 97% were African-American, equal proportions of men and women; senate workers in the DC area — five participants were Caucasian, two were African-American and six were female |
| **Intervention** | Researchers wanted to study the public health response to the anthrax attack in Washington, DC, and the perceptions of postal and senate workers about this response. |
| **Measurement Instrument:** | Focus groups |
| **Outcomes** | *Source of information:* 33 postal workers stated that the media was their main source of information regarding the anthrax attack. Other participants said that the U.S. Postal Service (USPS) management team disseminated some information. Senate workers said that internal communication methods were their main source of information.  *Attitudes towards information:* The majority of participants in both groups expressed frustration over the lack of information during the anthrax attack. Participants said the information was confusing and not disseminated in a timely manner. Participants with hearing impairments expressed difficulty in obtaining information.  *Attitudes toward sources of information:* Most participants were unhappy with the information that was provided by the Centers for Disease Control (CDC), the DC health department or by the USPS. Postal workers felt that the CDC did not disseminate information to them properly or in a timely manner because of their social class, while senate workers felt that the information provided by the CDC was inconsistent. |

| **Study** | Perez-Lugo (2004) |
| --- | --- |
| **Objective** | To highlight the media-audience relationship in terms of how natural disasters affect populations. |
| **Methods** | *Design:*  qualitative descriptive  *Selection:* residents of eight different communities in Mayagüez, Puerto Rico were selected as part of a convenience sample |
| **Participants** | *Sample:* (*N* = 37)  *Characteristics:* participants were from a region of Puerto Rico that was affected by Hurricane Georges – women (*n* = 23), men (*n* = 14), ages ranged from 21 to 88, average age of 51, retired (*n* = 8), homemakers (*n* = 6), college students (*n* = 4), medical doctors (*n* = 4), clerical workers (*n* = 5), teachers or college professors (*n* = 4), business owners (*n* = 4), lawyer (*n* = 1), unemployed (*n* = 1) |
| **Intervention** | Researchers conducted interviews to determine the role of the media in a community’s experience and perception of natural disasters. |
| **Measurement Instrument:** | Semi-structured interviews |
| **Outcomes** | *Role of the media:* Participants indicated that the media played a vital role  in the way that they learned about and coped with the disaster. The media was the primary source of information regarding the disaster. Participants also used the media as a means to deal with the emotional aspects of  the disaster. |
